# Implementation of synchronization of multi-fractional-order of chaotic neural networks with a variety of multi-time-delays: Studying the effect of double encryption for text encryption

**DOI:** 10.1371/journal.pone.0270402

**Published:** 2022-07-01

**Authors:** Fatin Nabila Abd Latiff, Wan Ainun Mior Othman

**Affiliations:** Institute of Mathematical Sciences, Faculty of Science, Universiti Malaya, Kuala Lumpur, Malaysia; Hodeidah University, YEMEN

## Abstract

This research proposes the idea of double encryption, which is the combination of chaos synchronization of non-identical multi-fractional-order neural networks with multi-time-delays (FONNSMD) and symmetric encryption. Symmetric encryption is well known to be outstanding in speed and accuracy but less effective. Therefore, to increase the strength of data protection effectively, we combine both methods where the secret keys are generated from the third part of the neural network systems (NNS) and used only once to encrypt and decrypt the message. In addition, a fractional-order Lyapunov direct function (FOLDF) is designed and implemented in sliding mode control systems (SMCS) to maintain the convergence of approximated synchronization errors. Finally, three examples are carried out to confirm the theoretical analysis and find which synchronization is achieved. Then the result is combined with symmetric encryption to increase the security of secure communication, and a numerical simulation verifies the method’s accuracy.

## 1. Introduction

Fractional-order calculus was established in the seventeenth century and was initially proposed by [1] as the extension of the differentiation function and integration function from integer to arbitrary order. The fractional is as old as the integer, although it was used mainly in mathematics; as a result, the study of the system with fractional-order has remained a prominent research area ever since. Fractional-order systems are more suited to simulating complex systems with self-similar forms and more complicated dynamical properties than integer-order systems. This is because fractional-order provides a more precise instrument [2–5], a well-suited technology for analyzing fractal dimension concerns, persistent memory, and chaotic behaviour [6, 7].

Experts have conducted ground-breaking research since L’Hospital and Leibniz initially introduced fractional calculus in 1675. These studies involve several branches of discipline and engineering, such as bioengineering, robots, heat conduction, electromagnetic waves, viscoelastic systems, and dielectric polarization [8–11]. Research has revealed that the fractional-order is able to portray the connection between input and output signals of NNS. Besides, the fractional-order has better memory and inheritance factors than the integer-order. It is possible to accurately model the neuron dynamics characteristics using fractional-order and NNS systems [12–16]. This combination is called fractional-order neural networks (FONNS), which has garnered tremendous attention from scholars, and some outstanding outcomes have been explored and published in [17–21]. The fact that FONNS have an endless amount of memory is also worth mentioning because it is believed that they will perform well in some functions such as parameter estimation because of their limitless memory. Considering these facts, incorporating a memory term into an NNS model is a significant enhancement that has been successfully applied to NNS. According to the literature [22–27], the authors have mentioned that chaotic behaviours can occur in a FONNS. Moreover, they emphasized the importance of establishing and analyzing mathematical models of FONNS because differentiation of fractional-order generates neurons with an essential and universal computation potential that can stipulate competent information processing. As a result, it is crucial and intriguing to investigate FONNS in their theoretical and practical applications.

Following the first success of Pecora and Carroll [28], the synchronization of FONNS has become a meaningful area in the research field. The term "synchronization of FONNS" refers to the fact that the state of FONNS nodes is primarily consistent with time. When two FONNS systems, the DSS and RSS, are coupled, both networks will act as excitation sources and gradually employ the control approach to eliminate the synchronization errors between the two FONNS systems. In addition, two FONNS must be synchronized so that the RSS output equals the DSS output asymptotically, which is not always possible. Aside from that, in [19–21], chaos control and synchronization based on FONNS were presented, which mainly relied on Laplace transformation theory and numerical simulations. The synchronization of chaotic systems has received much interest in the latest years due to the vast number of researchers who have developed various technologies in this field. One of the many applications of chaotic neural networks is secure communication.

Recent years have seen a resurgence in interest in FONNS-based modelling, owing to its tremendous advantages in controlling an issue and tackling complicated nonlinear system analysis problems. An increasing variety of synchronization systems have already been developed, including projective synchronization, anti-synchronization, complete synchronization, lag synchronization and other types of synchronization. Furthermore, we discovered that projective synchronization could accomplish faster communication with its proportional function. Most of the existing works by researchers are focused on the FONNS without delay [29–31], which we believe is incorrect. In fact, many complex systems are unable to avoid time-delay due to the nature of their systems [32, 33]. In every system, there is always some noise and disturbances that can significantly affect the dynamic features of the system, affecting its performance and, as a result, interfering with the synchronization’s output. Because of this, further exploration of the FONNS model with time delay becomes increasingly necessary to be examined in both its theoretical and practical implications [34]. Nowadays, different types of control systems based on FONNS have been presented in recent years to achieve synchronization; generalized projective control [35] as well as adaptive control [36], linear feedback control [37], and sliding mode control (SMC) [30, 38] were introduced, which are all based on NNS. In addition, SMC has many excellent characteristics, such as low perturbation and parameter disturbance sensitivity, fast response, and simplicity of implementation.

Due to the significant growth of communication technologies, cryptography plays a crucial part in personal email and computers, electronic fund transfers, and wireless networks. While these technologies have proliferated, users must accept the dangers associated with secure networks to benefit from the ease associated with secure network communication. The primary issues are mobile communication links, terminals, and confidentiality in authentication mechanisms [39–41]. Secure communication is not only about security and protecting people’s privacy but also about protecting people’s assets while performing exchanges over the network and about national defence security to protect the message’s and data’s secrets from data alteration and spying by hackers. Implementing encryption and decryption techniques is significant to overcoming the security problem of secure network communication.

Traditionally, cryptography has been used to keep the data by encrypting the accessible data into unreadable scrambled characters. Four primary characteristics needed in cryptography are authenticity, integrity, confidentiality, and usability [42–44]. Traditional cryptography algorithms are categorized into two types of cryptosystems: symmetric and asymmetric. Symmetric encryption is when the sender and receiver share the same key and can be designed to support high bandwidth throughout the system [45]. However, symmetric encryption has significant drawbacks, especially public key sharing. As a result, we designed an NNS chaotic encryption method that combines FONNSMD and symmetric encryption to strengthen the cryptosystem and prevent key distribution recurrence. NNS chaotic encryption [46–49] extensively uses chaos theory’s fundamental mixing properties and its high sensitivity to parameters and beginning values. Early communication in chaotic encryption algorithms (CEA) is classified into four types: a chaotic expansion [50], chaotic keying [51], chaotic masking [52], and chaotic parameter modulation [53]. Moreover, in classical CEA, the encryption speed is slowed, and its encryption efficiency is low. As a result, it is essential and worthwhile to study an effective NNS chaotic encryption method for communication security.

With the above motivations, our foremost aim in this paper is to combine integer-order and fractional-order multi-FONNSMD, which continues our previous work [34]. Our previous work has proven that synchronization is achieved with different values of fractional-order of time-delay. In comparison, this paper aims to establish that synchronization of multi-FONNSMD can be archived when we combine integer-order in DSS and fractional-order in RSS. However, to the best of the author’s knowledge, there are few results on the synchronization of fractional-order multi-FONNSMD systems and integer-order multi-FONNSMD systems, and we believe that the concept has never been studied. Furthermore, in this paper, we have compared the methods without delay, with time-delay, and time-varying delays. These three examples show that all three types of delayed systems can be achieved with a suitable parameter suggested. Besides, we analyze and introduce a combination of multi-FONNSMD and symmetric encryption called "Neural Network Symmetric Encryption" (NNSE) to form a stable private communication system. We called our system multi-FONNSMD because the fractional-order of the system will use a different order, and we used five different values of fractional-order and one value of integer-order.

Multi-FONNSMD synchronization can be accomplished with different DRS initial conditions with the specified values of parameters. It should also be noted that with several time delays, the conventional Lyapunov functional method (LFM) cannot be applied to multi-FONNSMD, but FOLDF stability theorems are more applicable. This combination is called the third generation of chaotic secure communication proposed by [54]. Their idea was to improve the system’s security even higher than the older generations. This generation is called a chaotic cryptosystem. Here, the NNSE technique is used to enhance the degree of protection. This generation has the highest security among the other chaotic secure communication system generations. Most secure communication uses this cryptosystem because of its advantages: it is fast sending of the message and its easy execution [55].

The four significant impacts of this paper are as follows:

1. First, a new controller based on SMC is created to achieve synchronization of multi-FONNSMD.
2. Second, based on the linear FOC system’s stability theorem, we present novel synchronization criteria for multi-FONNSMD, fractional-order and integer-order.
3. Third, a chaotic encryption approach based on NNS is being developed. This method combines multi-FONNSMD and symmetric encryption.
4. Fourth, some multi-FONNSMD with and without delays comparative results are shown.
5. Finally, our experiments will demonstrate a significant improvement over past work. They will provide sufficient confidence to assert that the findings in this paper are far less conventional and more general.

The rest of this work is organized in the following way: Section 2 contains several fundamental definitions, theorems, lemmas, and a summary of the model’s explanation. Section 3 includes the implementation of two multi-FONNSMD synchronization schemes. An introduction to the symmetry encryption algorithm is covered in Section 4, while in Section 5, we proposed a new NNSE algorithm. Section 6 presents the study results with three numerical examples to demonstrate the efficiency of the approaches provided. Lastly, a conclusion is drawn in section 7. Table 1 contains a list of acronyms used in this paper.

**Table 1:** Acronyms used in the article.

Acronyms	Description
CTEXT	Ciphertext
DSMC	Delayed sliding mode control
DSS	Drive systems
DRS	Drive-response system
EDSMC	Error delayed sliding mode control
FOC	Fractional order calculus
FOLDF	Fractional-order Lyapunov direct function
FONNS	Fractional-order neural networks
FONNSMD	Fractional-order neural networks multi delayed
G-L	Grünwald–Letnikov
LFM	Lyapunov functional method
NNSE	Neural networks symmetric encryption
NNS	Neural networks
PTEXT	Plaintext
PKEY	Private keys
RSS	Response systems
R-L	Riemann–Liouville
SCA	Symmetric cryptographic algorithms
SKEY	Secret keys
SMCS	Sliding mode control system

## 2. Description of the model and the fundamental definitions of fractional-order derivative

Caputo, G-L, and R-L derivatives are three popular derivatives that serve as models for the fractional derivatives definitions. Improvements to the G-L allowed for the foundation of the Caputo and R-L. Between the Caputo derivative and the R-L derivative, the Caputo derivative is the most frequently used by researchers. The most significant advantage of the Caputo derivative is that it simply needs initial conditions expressed in conditions of integer-order derivatives, which describe elements of physical situations that are well recognized, making it more relevant to real-world problems. Another advantage of the Caputo derivative is that it is valid for homogeneous and non-homogeneous initial circumstances, which is not the case with the R-L derivative. In contrast, the R-L derivative is only valid for homogeneous initial conditions to solve initial value issues.

The disadvantages of R-L’s derivatives include the fact that the Laplace transform has fractional derivatives and that acquiring the initial conditions of the fractional derivatives is a time-consuming process. Furthermore, the physical and geometric significance of the fractional derivatives is not entirely comprehensible to us. However, the Caputo derivatives can solve this weakness. As a result, the Caputo derivatives are frequently employed in science and technology applications.

**Notations** Throughout this paper, the complex number space is denoted as the notation C. The real number space is denoted as the symbol R. In contrast, the space of n-dimensional Euclidean is denoted as R^n,^ and the sign function is denoted as a sign (·). Then let u > 0, C([–u, 0], R^n^) represent the group of continuous functions from [–u, 0] to R^n^.

**Definition 1**. [1] *Let the order be α*_1_ *and a positive integer* n. *Then α*_1_ > 0, *such that* $n-1<\alpha_{1}\leq n$. *The R-L fractional derivative of an* f(t) *is represented as*:

$$O_{x}^{\alpha_{1}}f\left(t\right)=\frac{{\text{d}}^{n}}{\text{d}t^{\text{n}}}j_{x}^{n-\alpha_{1}}f\left(t\right),$$

*Furthermore, the R-L fractional integral is represented as*:

$$\text{I}f\left(t\right)=\frac{1}{\Gamma \left(\alpha_{1}\right)}\int_{0}^{t}{\left(t-\text{u}\right)}^{\alpha_{1}-1}f\left(\text{u}\right)\text{du},$$

**Definition 2**. [1] *Let the order be α*_1_, *and a positive integer* n. *Then n* − 1 < *α*_1_ ≤ *n*. *The expression represents the Caputo fractional derivative of an* f(t):

$$D_{t}^{\alpha_{1}}f\left(t\right)=\frac{1}{\Gamma \left(n-\alpha_{1}\right)}{\int^{\;}}_{0}^{t}{\left(t-\text{u}\right)}^{n-\alpha_{1}-1}f^{n}\left(\text{u}\right)\text{du},$$

*When* 0 < *α*_1_ < 1,

$$D_{t}^{\alpha_{1}}f\left(t\right)=\frac{1}{\Gamma \left(1-\alpha_{1}\right)}\int_{0}^{t}{\left(t-\text{u}\right)}^{-\alpha_{1}}f^{\prime }\left(\text{u}\right)\text{du}$$

*Let the order be α*_1_ *and with α*_1_ > 0, *and the Caputo fractional integral of a function* f(t), *be represented by the expression*:

$${\text{I}}_{t}^{\alpha_{1}}f\left(t\right)=\frac{1}{\Gamma \left(\alpha_{1}\right)}{\int^{\;}}_{0}^{t}{\left(t-\text{u}\right)}^{\alpha_{1}-1}f\left(\text{u}\right)\text{du},$$

Γ(·) is the Gamma function, $\Gamma \left(\alpha_{1}\right)=\int_{0}^{+\infty }e^{-t}t^{\alpha_{1}-1}dt$

**Definition 3**. [1] **${Ε}_{\alpha_{1}}\left(x\right)$** *represents the function of Mittag-Leffler with one parameter, where α*_1_ > 0, *and* xϵC *is described as*

$${\text{E}}_{\alpha_{1}}\left(x\right)=\overset{\infty }{\underset{\text{k}=0}{\sum^{\;}}}\frac{x^{\text{k}}}{\Gamma \left({\text{k}\alpha }_{1}+1\right)}$$

${Ε}_{\alpha_{1},\alpha_{2}}\left(x\right)$ *denotes the function of Mittag-Leffler with two parameters*, *where α*_1_ > 0, *α*_2_ > 0 *is defined as*

$${\text{E}}_{\alpha_{1},\alpha_{2}}\left(x\right)=\overset{\infty }{\underset{\text{k}=0}{\sum^{\;}}}\frac{x^{\text{k}}}{\Gamma \left({\text{k}\alpha }_{1}+\alpha_{2}\right)}$$

*When α*_2_ = 1, *one has* ${Ε\;}_{\alpha_{1}}\left(x\right)={Ε}_{\alpha_{1},1}\left(x\right)$, *and when α*_1_ = 1, *α*_2_ = 1, *one further has E*_1,1_(*x*) = *e*^*x*^

We study the following NNS system in this paper, which is called multi-FONNSMD as the drive systems (DSS) where 0 < α_1_ < 1, and denoted it as

$$D^{\alpha_{1}}x_{i}\left(t\right)=-{\text{a}}_{i}x_{i}\left(t\right)+\overset{\text{n}}{\underset{j=1}{\sum^{\;}}}{\text{b}}_{ij}f_{j}\left(x_{j}\left(t\right)\right)+\overset{\text{n}}{\underset{j=1}{\sum^{\;}}}{\text{c}}_{ij}{\text{f}}_{j}\left(x_{j}\left(t-\pi \tau \right)\right)+{\text{U}}_{i}$$ (1)

Alternatively, the vector form:

$$D^{\alpha_{1}}x\left(t\right)=-\text{A}x\left(t\right)+\text{B}f\left(x\left(t\right)\right)+\text{C}f\left(x\left(t-\pi \tau \right)\right)+\text{U}$$

The parameter $a_{i}>0$ is the ith neuron’s self-inhibition, and the pseudo-state variable of the DSS for the ith neuron is represented by the parameter $x_{i}\left(t\right)$. Another two parameters, ${\text{b}}_{ij}$ and ${\text{c}}_{ij}$, represent the relationship between the ith neuron and ith neuron at a time interval of t and t−πτ, in which π is a positive integer. Let n be the number of neurons in a multi-FONNSMD and i,j=1,2,...,n. Note that πτ > 0 represents the delayed time. For the ith neuron’s activation function output, we denoted it as ${\text{f}}_{j}\left(x_{j}\left(t\right)\right)$ and ${\text{f}}_{j}\left(x_{j}\left(t-\pi \tau \right)\right)$ at time t and t−πτ, and finally, the external input of the ith neuron is represented as ${\text{U}}_{i}$.

The corresponding response system (RSS), where 0 < α_2_ < 1, is denoted as

$$D^{\alpha_{2}}{\text{y}}_{i}\left(t\right)=-{\text{p}}_{i}{\text{y}}_{i}\left(t\right)+\overset{n}{\underset{j=1}{\sum }}{\text{q}}_{ij}g_{j}\left({\text{y}}_{j}\left(t\right)\right)+\overset{n}{\underset{j=1}{\sum }}{\text{r}}_{ij}g_{j}\left({\text{y}}_{j}\left(t-\pi \tau \right)\right)+{\text{V}}_{i}+{\text{S}}_{{\text{C}}_{\text{i}}}\left(t\right)$$ (2)

Alternatively, the vector form:

$$D^{\alpha_{2}}\text{y}\left(t\right)=-\text{Py}\left(t\right)+\text{Q}g\left(\text{y}\left(t\right)\right)+\text{R}g\left(\text{y}\left(t-\pi \tau \right)\right)+\text{V}+{\text{S}}_{\text{C}}\left(t\right)$$

$\text{A},\text{B},\text{C},\text{P},\text{Q},\text{R},\;M_{1}$ and $M_{2}$ denote the constant matrices.

The parameter ${\text{p}}_{i}>0$ is the ith neuron’s self-inhibition, and the pseudo-state variable of the DSS for the ith neuron is represented by the parameter ${\text{y}}_{i}\left(t\right)$. Another two parameters, ${\text{q}}_{ij}$ and ${\text{r}}_{ij}$ represent the relationship between the ith neuron and the ith neuron at a time interval of t and t−πτ, in which π is a positive integer. Let n be the number of neurons in a FONNSMD and i,j=1,2,...,n. We noted that $\tau_{i}>0$ represents the delayed time. For the ith neuron’s activation function output, we denoted it as $g_{j}\left({\text{y}}_{j}\left(t\right)\right)$ and $g_{\;j}\left({\text{y}}_{\;j}\left(t-\pi \tau \right)\right)$ at a time t and t−πτ, and the external input of the ith neuron is denoted as ${\text{V}}_{i}$. Finally, ${\text{S}}_{{\text{C}}_{\text{i}}}\left(t\right)=M_{1}x\left(t\right)+M_{2}x\left(t-\pi \tau \right)$ represents the SMCS.

Another definition, theorems, and lemmas are given in the next part for further explanation.

**Lemma 4**. [56] *Let $t\;\epsilon \;\left[\;t_{0}\;,\;\infty \right),\;\;\;\Theta \;\epsilon \;PC\left[\;\left[\;-\tau ,\;0\right],\;\;\;R^{n}\right],\;\;\varphi \;\epsilon \;R$ is a constant.*

*Assume that for function V, the Caputo upper right-hand derivative of order,* 0 < *α*_1_ < 1 *with the following inequality holds*:

$$D_{+}^{\alpha_{1}}V\left[t,\Theta \left(0\right)\right]\leq \varphi V\left[t,\Theta \left(0\right)\right]$$

Every time $V\left[t+\;r,\Theta \left(\;r\right)\right]\leq V\left[t,\Theta \left(0\right)\right]$ for −τ≤r≤0.

Then $\underset{\tau \leq \;r\leq 0}{sup}V\;\left[t_{0}+s,\;\Theta_{0}\;\left(\;r\right)\right]\;\leq x_{0}$ denotes $V\left[t,\Theta \left(t\right)\right]\leq V\left[t_{0},\Theta \left(t_{0}\right)\right]{Ε}_{\alpha_{1}}\left[\varphi {\left(t-t_{0}\right)}^{\alpha_{1}}\right]$ *with* $t\;\epsilon \;\left[\;t_{0}\;,\;\infty \right)$.

**Definition 5**. [36] *Let ϖ be a nonzero constant. Assume there exists* $x\left(t\right)$ *and* $y\left(t\right)$ *such that any two results with distinct initial values of* Eqs (1) and (3) *have the following*:

$$\underset{\text{t}\to +\infty }{\text{lim}}||\text{y}\left(t\right)-\varpi x\left(t\right)||=0$$ (3)

As a result, both the DSS (1) and the RSS (2) can achieve projective synchronization, where ||·|| represents the Euclidean 1-norm of a vector.

**Lemma 6**. [57] *Let* 0 < *α*_1_ < 1 *with the function $x\left(t\right)\;\epsilon \;R$ be a continuous differentiable vector-value. Therefore, at every given time $t\geq t_{0}$, one has the following:*

$$D^{\alpha_{1}}x^{\text{T}}\left(t\right)\;x\left(t\right)\leq 2x^{\text{T}}\left(t\right)D^{\alpha_{1}}x\left(t\right)$$ (4)

**Definition 7**. [58] *Let α*_1_ *ϵ* (0, 1), *ϖ* > 0, *γ* > 0 *with $t_{0}$ representing the initial instant. $R\left(0\right)=0$*, *and $R\left(x\right)\geq 0$ then $R\left(x\right)$ is locally Lipschitz on xϵR about the constant $R_{0}$. The Mittag–Leffler* Eq (1) *is stable if*

$$||\;x\left(t\right)||\leq {\left\{R\left(x\left(t_{0}\right)\right){\text{E}}_{\alpha_{1}}{\left(-\varpi \left(t-t_{0}\right)\right)}^{\alpha_{1}}\right\}}^{\gamma }$$

**Lemma 8**. [59] *If $f\left(t\right),\;\;g\left(t\right)\;\epsilon \;C^{1}\;\left[\;t_{0}\;,\;b\right]$, then*

1.
$D^{-\alpha_{1}}D^{-\alpha_{2}}f\left(t\right)=D^{-\alpha_{1}-\alpha_{2}}f\left(t\right),\;\alpha_{1},\;\alpha_{2}\geq 0;\;$

2. $D^{\alpha_{1}}D^{-\alpha_{1}}f(t)=f(t),\;\alpha_{1}\geq 0$;
3. $D^{-\alpha_{1}}D^{\alpha_{1}}f(t)=f(t)\;-\sum_{k=0}^{n\;-1}\frac{t^{k}}{k!}f^{k}(0),\;\alpha_{1}\geq 0$.
4. $D^{\alpha_{1}}(v_{1}f(t)+v_{2}\;g(t))=v_{1}\;D^{\alpha_{1}}f(t)\;+\;v_{2}{\;D}^{\alpha_{1}}g(t)$, v_1_ and v_2_ are constants.
5. $D^{\alpha_{1}}c=0,\;c$ is a constant.

**Lemma 9**. [60] *If function* $f\left(t\right)\;\epsilon \;C^{1}\;\left(\left[0,\;\infty \;\right),\;R\right)$ *is differentiable, then the following inequality holds* $D^{\alpha_{1}}\left|f(t)\right|\leq sign\left(f\left(t\right)\right)D^{\alpha_{1}}\;f\left(t\right).$

**Lemma 10**. [61] *let* x=0 *as an equilibrium point of DSS* (1). *Assume that there exists a Lyapunov function* V(t,x) *and class-K functions h*_*i*_ (*i* = 1, 2, 3) *satisfying*:

$$h_{1}\left(\left|\left|x\right|\right|\right)\leq V\left(t,\;x\right)\leq h_{2}\left(\left|\left|x\right|\right|\right)$$ (5)

$$D^{\alpha_{1}}V\left(t,\;x\right)\leq -h_{3}\left(\left|\left|x\right|\right|\right)$$ (6)

*where α*_1_ ∈ (0,1). *Hence*, *the equilibrium point of the DSS* (1) *is asymptotically stable*.

**Assumption (H)**. Assume that there exist real numbers $p_{i}^{-},\;p_{i}^{+},\;q_{i}^{-}$ and $q_{i}^{+}$ such that for any constants u≠v∈R, the following conditions always hold for all *i* = 1, 2, …, *n*

$$p_{i}^{-}\;\leq \frac{f_{i}\;(u)\;-f_{i}\;(v)\;}{u-\;v}\leq \;p_{i}^{+}\;\text{and}\;q_{i}^{-}\;\leq \frac{g_{i}\;(u)\;-g_{i}\;(v)\;}{u-\;v}\leq \;q_{i}^{+}$$

## 3. Synchronization scheme of multi-FONNSMD

The synchronization of two multi-FONNSMD, namely DSS (1) and RSS (3), is achieved in this section by designing a suitable control system. Synchronization dynamic errors are expressed as $S_{E_{i}}\left(t\right)=y_{i}(t)-\varpi x_{i}(t)$. Where i,j,n,u,π=1,2,…,n and ϖ is the projective coefficient.

Using the DSS (1) and the RSS (2) as a foundation, we proposed the delayed sliding mode control (DSMC), $S_{C_{i}}\left(t\right)$ as the following functions:

$$\begin{array}{l} {\text{S}}_{{\text{C}}_{\text{i}}}\left(t\right)=-\left({\text{a}}_{i}+\left(M_{1_{j\text{k}}}\right)\cdot \left(M_{2_{\text{k}i}}\right)-{\text{p}}_{i}\right){\text{S}}_{{\text{E}}_{i}}\left(t\right)-\left(M_{1_{j\text{k}}}\right)\cdot \left(M_{3_{\text{n}i}}\right){\text{S}}_{{\text{E}}_{i}}\left(t-\pi \tau \right)-\;\varpi \left({\text{a}}_{i}-{\text{p}}_{i}\right)x_{i}\left(t\right)\\ \;-\overset{\text{n}}{\underset{j=1}{\sum }}{\text{q}}_{ij}\left[g_{j}\left(\varpi x_{j}\left(t\right)\right)\right]-\overset{\text{n}}{\underset{j=1}{\sum }}{\text{r}}_{ij}\left[g_{j}\left(\varpi x_{j}\left(t-\pi \tau \right)\right)\right]\\ \;+\varpi \overset{\text{n}}{\underset{j=1}{\sum }}{\text{b}}_{ij}\left[{\text{f}}_{j}\left(x_{j}\left(t\right)\right)\right]+\varpi \overset{\text{n}}{\underset{j=1}{\sum }}{\text{c}}_{ij}\left[{\text{f}}_{j}\left(x_{j}\left(t-\pi \tau \right)\right)\right]+\varpi {\text{U}}_{i}-{\text{V}}_{i} \end{array}$$ (7)

From (1), (2) and (7), the system of error delayed, $D^{\alpha }S_{E}\left(t\right)$ is derived as follows:

$$\begin{array}{l} D^{\alpha }{\text{S}}_{{\text{E}}_{i}}\left(t\right)={\text{p}}_{i}{\text{S}}_{{\text{E}}_{i}}\left(t\right)+\overset{\text{n}}{\underset{j=1}{\sum }}{\text{q}}_{ij}\left[g_{j}\left({\text{y}}_{j}\left(t\right)\right)-g_{j}\left(\varpi x_{j}\left(t\right)\right)\right]+\;\overset{\text{n}}{\underset{j=1}{\sum }}{\text{r}}_{ij}\left[g_{j}\left({\text{y}}_{j}\left(t-\tau_{i}\right)\right)-g_{j}\left(\varpi x_{j}\left(t-\pi \tau \right)\right)\right]\\ \;+\varpi \left({\text{a}}_{i}-{\text{p}}_{i}\right)x_{i}\left(t\right)+\overset{\text{n}}{\underset{j=1}{\sum }}{\text{q}}_{ij}\left[g_{j}\left(\varpi x_{j}\left(t\right)\right)\right]+\overset{\text{n}}{\underset{j=1}{\sum }}{\text{r}}_{ij}\left[g_{j}\left(\varpi x_{j}\left(t-\pi \tau \right)\right)\right]\\ \;-\varpi \overset{\text{n}}{\underset{j=1}{\sum }}{\text{b}}_{ij}\left[{\text{f}}_{j}\left(x_{j}\left(t\right)\right)\right]-\varpi \overset{\text{n}}{\underset{j=1}{\sum }}{\text{c}}_{ij}\left[{\text{f}}_{j}\left(x_{j}\left(t-\pi \tau \right)\right)\right]-\varpi {\text{U}}_{i}+{\text{V}}_{i}+{\text{S}}_{{\text{C}}_{\text{i}}}\left(t\right) \end{array}$$ (8)

By utilizing Lemma 8,

$$\begin{array}{l} {\text{S}}_{{\text{E}}_{i}}\left(t\right)={\text{S}}_{{\text{E}}_{i}}\left(0\right)+\;D^{-\alpha }[{\text{p}}_{i}{\text{S}}_{{\text{E}}_{i}}\left(t\right)+\overset{\text{n}}{\underset{j=1}{\sum }}{\text{q}}_{ij}\left[g_{j}\left({\text{y}}_{j}\left(t\right)\right)-g_{j}\left(\varpi x_{j}\left(t\right)\right)\right]\\ \;+\overset{\text{n}}{\underset{j=1}{\sum }}{\text{r}}_{ij}\left[g_{j}\left({\text{y}}_{j}\left(t-\tau_{i}\right)\right)-g_{j}\left(\varpi x_{j}\left(t-\pi \tau \right)\right)\right]+\varpi \left({\text{a}}_{i}-{\text{p}}_{i}\right)x_{i}\left(t\right)\\ \;+\overset{\text{n}}{\underset{j=1}{\sum }}{\text{q}}_{ij}\left[g_{j}\left(\varpi x_{j}\left(t\right)\right)\right]+\overset{\text{n}}{\underset{j=1}{\sum }}{\text{r}}_{ij}\left[g_{j}\left(\varpi x_{j}\left(t-\pi \tau \right)\right)\right]-\;\varpi \overset{\text{n}}{\underset{j=1}{\sum }}{\text{b}}_{ij}\left[{\text{f}}_{j}\left(x_{j}\left(t\right)\right)\right]\\ \;-\varpi \overset{\text{n}}{\underset{j=1}{\sum }}{\text{c}}_{ij}\left[{\text{f}}_{j}\left(x_{j}\left(t-\pi \tau \right)\right)\right]\;-\varpi {\text{U}}_{i}+{\text{V}}_{i}+{\text{S}}_{{\text{C}}_{\text{i}}}\left(t\right)] \end{array}$$ (9)

From the synchronization error (9), we represented the delayed sliding switching surface (DSSS) as:

$$\begin{array}{l} {\text{S}}_{{\text{S}}_{\text{i}}}\left(t\right)={\text{S}}_{{\text{E}}_{i}}\left(t\right)+D^{-\alpha }\left[\left(M_{1_{j\text{k}}}\right)\left(\left(M_{2_{\text{k}i}}\right)\cdot {\text{S}}_{{\text{E}}_{i}}\left(t\right)+\left(M_{3_{\text{n}i}}\right)\cdot {\text{S}}_{{\text{E}}_{i}}\left(t-\pi \tau \right)-\varpi {\text{S}}_{\text{C}}\left(t\right)\right)\right]\\ \;-D^{-\alpha }\;[-{\text{a}}_{i}{\text{S}}_{{\text{E}}_{i}}\left(t\right)+\overset{\text{n}}{\underset{j=1}{\sum }}{\text{q}}_{ij}\left[g_{j}\left({\text{y}}_{j}\left(t\right)\right)-g_{j}\left(\varpi x_{j}\left(t\right)\right)\right]\\ \;+\overset{\text{n}}{\underset{j=1}{\sum }}{\text{r}}_{ij}\left[g_{j}\left({\text{y}}_{j}\left(t-\tau_{i}\right)\right)-g_{j}\left(\varpi x_{j}\left(t-\pi \tau \right)\right)\right] \end{array}$$ (10)

Where gain matrix, ${\left({M_{1}}_{jk}\right)}_{n\times u}$ is chosen appropriately. Then, we substitute Eq (9) into Eq (10), and one finds that:

$$\begin{array}{l} {\text{S}}_{{\text{S}}_{\text{i}}}\left(t\right)={\text{S}}_{{\text{E}}_{i}}\left(0\right)+D^{-\alpha }[\left({\text{a}}_{i}+\left(M_{1_{j\text{k}}}\right)\cdot \left(M_{2_{\text{k}i}}\right)-{\text{p}}_{i}\right){\text{S}}_{{\text{E}}_{i}}\left(t\right)+\left(M_{1_{j\text{k}}}\right)\cdot \left(M_{3_{\text{n}i}}\right){\text{S}}_{{\text{E}}_{i}}\left(t-\pi \tau \right)\\ \;+\varpi \left({\text{a}}_{i}-{\text{p}}_{i}\right)x_{i}\left(t\right)+\overset{\text{n}}{\underset{j=1}{\sum }}{\text{q}}_{ij}\left[g_{j}\left(\varpi x_{j}\left(t\right)\right)\right]+\overset{\text{n}}{\underset{j=1}{\sum }}{\text{r}}_{ij}\left[g_{j}\left(\varpi x_{j}\left(t-\pi \tau \right)\right)\right]\\ \;-\varpi \overset{\text{n}}{\underset{j=1}{\sum }}{\text{b}}_{ij}\left[{\text{f}}_{j}\left(x_{j}\left(t\right)\right)\right]-\varpi \overset{\text{n}}{\underset{j=1}{\sum }}{\text{c}}_{ij}\left[{\text{f}}_{j}\left(x_{j}\left(t-\pi \tau \right)\right)\right]-\varpi {\text{U}}_{i}+{\text{V}}_{i}+{\text{S}}_{{\text{C}}_{i}}\left(t\right)] \end{array}$$ (11)

Subsequently, it follows from the Eq (11) that

$$\begin{array}{l} D^{\alpha }{\text{S}}_{{\text{S}}_{\text{i}}}\left(t\right)=D^{\alpha }{\text{S}}_{{\text{E}}_{i}}\left(0\right)\\ \;\;\;+D^{\alpha }D^{-\alpha }[\left({\text{a}}_{i}+\left(M_{1_{j\text{k}}}\right)\cdot \left(M_{2_{\text{k}i}}\right)-{\text{p}}_{i}\right){\text{S}}_{{\text{E}}_{i}}\left(t\right)+\left(M_{1_{j\text{k}}}\right)\cdot \left(M_{3_{\text{n}i}}\right){\text{S}}_{{\text{E}}_{i}}\left(t-\pi \tau \right)\\ \;\;+\varpi \left({\text{a}}_{i}-{\text{p}}_{i}\right)x_{i}\left(t\right)+\overset{\text{n}}{\underset{j=1}{\sum }}{\text{q}}_{ij}\left[g_{j}\left(\varpi x_{j}\left(t\right)\right)\right]+\overset{\text{n}}{\underset{j=1}{\sum }}{\text{r}}_{ij}\left[g_{j}\left(\varpi x_{j}\left(t-\pi \tau \right)\right)\right]\\ \;\;\;\;-\varpi \overset{\text{n}}{\underset{j=1}{\sum }}{\text{b}}_{ij}\left[{\text{f}}_{j}\left(x_{j}\left(t\right)\right)\right]-\varpi \overset{\text{n}}{\underset{j=1}{\sum }}{\text{c}}_{ij}\left[{\text{f}}_{j}\left(x_{j}\left(t-\pi \tau \right)\right)\right]-\varpi {\text{U}}_{i}+{\text{V}}_{i}+{\text{S}}_{{\text{C}}_{\text{i}}}\left(t\right)] \end{array}$$

$$\begin{array}{l} =\left({\text{a}}_{i}+\left(M_{1_{j\text{k}}}\right)\cdot \left(M_{2_{\text{k}i}}\right)-{\text{p}}_{i}\right){\text{S}}_{{\text{E}}_{i}}\left(t\right)+\left(M_{1_{j\text{k}}}\right)\cdot \left(M_{3_{\text{n}i}}\right){\text{S}}_{{\text{E}}_{i}}\left(t-\pi \tau \right)+\varpi \left({\text{a}}_{i}-{\text{p}}_{i}\right)x_{i}\left(t\right)\\ \;\;\;+\overset{\text{n}}{\underset{j=1}{\sum }}{\text{q}}_{ij}\left[g_{j}\left(\varpi x_{j}\left(t\right)\right)\right]+\overset{\text{n}}{\underset{j=1}{\sum }}{\text{r}}_{ij}\left[g_{j}\left(\varpi x_{j}\left(t-\pi \tau \right)\right)\right]\\ \;\;-\varpi \overset{\text{n}}{\underset{j=1}{\sum }}{\text{b}}_{ij}\left[{\text{f}}_{j}\left(x_{j}\left(t\right)\right)\right]-\varpi \overset{\text{n}}{\underset{j=1}{\sum }}{\text{c}}_{ij}\left[{\text{f}}_{j}\left(x_{j}\left(t-\pi \tau \right)\right)\right]-\varpi {\text{U}}_{i}+{\text{V}}_{i}+{\text{S}}_{{\text{C}}_{\text{i}}}\left(t\right) \end{array}$$ (12)

Based on the theory of DSMC, DSSS and its derivative must meet $S_{E}\left(t\right)=0$ and $\dot{S_{E}}\left(t\right)=0$. Also, we can acquire $\dot{S_{E}}\left(t\right)=D^{1-\alpha }D^{\alpha }S_{E}\left(t\right)$ by applying Lemma 8. Therefore, $\dot{S_{E}}\left(t\right)=0$ is equal to $D^{\alpha }S_{E}\left(t\right)=0$. Hence, the result is ${D^{\alpha }S}_{S_{i}}\left(t\right)=0,$ and we prove that DSMC is an Eq (7).

Now Eqs (7) and (8) are combined. Then $D^{\alpha }S_{E}\left(t\right)$ can be defined by

$$\begin{array}{l} D^{\alpha }{\text{S}}_{\text{E}}\left(t\right)=\left[-{\text{a}}_{i}+M_{1}\cdot M_{2}\right]{\text{S}}_{{\text{E}}_{i}}\left(t\right)-\left[M_{1}\cdot M_{3}\right]{\text{S}}_{{\text{E}}_{i}}\left(t-\pi \tau \right)+\overset{\text{n}}{\underset{j=1}{\sum }}{\text{q}}_{ij}\left[g_{j}\left({\text{y}}_{j}\left(t\right)\right)-{\text{g}}_{j}\left(\varpi x_{j}\left(t\right)\right)\right]\\ \;+\overset{\text{n}}{\underset{j=1}{\sum }}{\text{r}}_{ij}\left[g_{j}\left({\text{y}}_{j}\left(t-\pi \tau \right)\right)-g_{j}\left(\varpi x_{j}\left(t-\pi \tau \right)\right)\right] \end{array}$$ (13)

Then, Eq (13) is transformed as follows:

$$\begin{array}{l} D^{\alpha }{\text{S}}_{{\text{E}}_{\text{i}}}\left(t\right)=-{\text{a}}_{i}{\text{S}}_{{\text{E}}_{i}}\left(t\right)-\left[\overset{\text{n}}{\underset{j=1}{\sum }}\overset{\text{u}}{\underset{\text{k}=1}{\sum }}\left(M_{1_{j\text{k}}}\right)\left(M_{2_{\text{k}i}}\right)\right]\;{\text{S}}_{{\text{E}}_{\text{j}}}\left(t\right)-\left[\overset{\text{n}}{\underset{j=1}{\sum }}\overset{\text{u}}{\underset{\text{k}=1}{\sum }}\left(M_{1_{j\text{k}}}\right)\left(M_{3_{\text{k}i}}\right)\right]\;{\text{S}}_{{\text{E}}_{j}}\left(t-\pi \tau \right)\\ \;+\overset{\text{n}}{\underset{j=1}{\sum }}{\text{q}}_{ij}\left[g_{j}\left({\text{y}}_{j}\left(t\right)\right)-g_{j}\left(\varpi x_{j}\left(t\right)\right)\right]+\overset{\text{n}}{\underset{j=1}{\sum }}{\text{r}}_{ij}\left[g_{j}\left({\text{y}}_{j}\left(t-\pi \tau \right)\right)-g_{j}\left(\varpi x_{j}\left(t-\pi \tau \right)\right)\right] \end{array}$$ (14)

**Theorem 11**. [62] Assume that $u_{i}$ and $k_{i}$ are positive constants, and that $u_{i}=\text{max}\left\{\left|u_{i}^{-}\right|,\left|u_{i}^{+}\right|\right\},\;k_{i}=\text{max}\left\{\left|k_{i}^{-}\right|,\left|k_{i}^{+}\right|\right\}$. Then the three matrices: ${\left({M_{1}}_{jk}\right)}_{n\times u},\;{\left({M_{2}}_{ki}\right)}_{n\times u}$, and ${\left({M_{3}}_{ki}\right)}_{u\times n}$. Suppose that assumption (**H**) holds and then;

$$\left\{\begin{array}{c} \Psi_{1}≔\underset{1\leq i\leq \text{n}}{\text{min}}\left[{\text{a}}_{i}-\overset{\text{n}}{\underset{j=1}{\sum }}\left[\overset{\text{u}}{\underset{\text{k}=1}{\sum }}\left|\left(M_{1_{j\text{k}}}\right)\left(M_{2_{\text{k}i}}\right)\right|\right]-\overset{\text{n}}{\underset{j=1}{\sum }}\left|{\text{q}}_{ji}\right|{\text{u}}_{i}\right]>0\\ \Psi_{2}≔\underset{1\leq i\leq \text{n}}{\text{max}}\left[\overset{\text{n}}{\underset{j=1}{\sum }}\left[\overset{\text{u}}{\underset{\text{k}=1}{\sum }}\left|\left(M_{1_{j\text{k}}}\right)\left(M_{3_{\text{k}i}}\right)\right|\right]\;+\overset{\text{n}}{\underset{j=1}{\sum }}\left|{\text{r}}_{ji}\right|{\text{k}}_{i}\;\right]>0\\ \Psi_{1}-\Psi_{2}>0 \end{array}\right.$$ (15)

Then the Eq (13) is Mittag-Leffler stable.

**Proof**. Corresponding to Eq (15), Theorem 11 and assumption **(H)** assume that we have

$${\text{u}}_{j}^{-}\leq \frac{{\text{f}}_{j}\left[{\text{y}}_{j}\left(t\right)\right]-{\text{f}}_{j}\left[\varpi x_{j}\left(t\right)\right]}{\left[{\text{y}}_{j}\left(t\right)\right]-\left[\varpi x_{j}\left(t\right)\right]}\leq {\text{u}}_{j}^{+}$$

and

$${\text{k}}_{j}^{-}\leq \frac{g_{j}\left[{\text{y}}_{j}\left(t-\pi \tau \right)\right]-g_{j}\left[\varpi x_{j}\left(t-\pi \tau \right)\right]}{\left[{\text{y}}_{j}\left(t-\pi \tau \right)\right]-\left[\varpi x_{j}\left(t-\pi \tau \right)\right]}\leq {\text{k}}_{j}^{+}$$

Next, we obtain that

$$\left\{ \begin{array}{c} \left|{\text{f}}_{j}\left[{\text{y}}_{j}\left(t\right)\right]-{\text{f}}_{j}\left[\varpi x_{j}\left(t\right)\right]\right|\leq {\text{u}}_{j}\left|\left({\text{y}}_{j}\left(t\right)\right)-\left(\varpi x_{j}\left(t\right)\right)\right|\\ \left|g_{j}\left[{\text{y}}_{j}\left(t-\pi \tau \right)\right]-g_{j}\left[\varpi x_{j}\left(t-\pi \tau \right)\right]\right|\leq {\text{k}}_{j}\left|\left({\text{y}}_{j}\left(t-\pi \tau \right)\right)-\left(\varpi x_{j}\left(t-\pi \tau \right)\right)\right| \end{array}\right.$$ (16)

The Lyapunov function, which has been constructed and demonstrated, is as follows:

$$V\left(t\right)=\overset{\text{n}}{\underset{i=1}{\sum }}\left|{\text{S}}_{{\text{E}}_{i}}\left(t\right)\right|$$ (17)

From Lemma 9 and 10 by allowing the right upper $D_{+}^{\alpha }\left[S_{E}\left(t\right)\right]$ along with the trajectory Eq (17). We expressed the Lyapunov function with the following:

$$\begin{array}{ll} D_{+}^{\alpha }V\left[{\text{S}}_{{\text{E}}_{i}}\left(t\right)\right] & =\overset{\text{n}}{\underset{i=1}{\sum }}D_{+}^{\alpha }\left|{\text{S}}_{{\text{E}}_{i}}\left(t\right)\right|\\ & \leq \overset{\text{n}}{\underset{i=1}{\sum }}\text{sign}\left[{\text{S}}_{{\text{E}}_{i}}\left(t\right)\right]{\text{D}}_{+}^{\alpha }{\text{S}}_{{\text{E}}_{i}}\left(t\right) \end{array}$$

$$\begin{array}{l} =\overset{\text{n}}{\underset{i=1}{\sum }}\text{sign}\left[{\text{S}}_{{\text{E}}_{i}}\left(t\right)\right][-{\text{a}}_{i}{\text{S}}_{{\text{E}}_{i}}\left(t\right)-\left[\overset{\text{n}}{\underset{j=1}{\sum }}\overset{\text{u}}{\underset{\text{k}=1}{\sum }}\left(M_{1_{j\text{k}}}\right)\left(M_{2_{\text{k}i}}\right)\right]{\text{S}}_{{\text{E}}_{\text{j}}}\left(t\right)\\ \;-\left[\overset{\text{n}}{\underset{j=1}{\sum }}\overset{\text{u}}{\underset{\text{k}=1}{\sum }}\left(M_{1_{j\text{k}}}\right)\left(M_{3_{\text{k}i}}\right)\right]{\text{S}}_{{\text{E}}_{j}}\left(t-\pi \tau \right)\\ \;+\overset{\text{n}}{\underset{j=1}{\sum }}{\text{q}}_{ij}\left[g_{j}\left({\text{y}}_{j}\left(t\right)\right)-g_{j}\left(\varpi x_{j}\left(t\right)\right)\right]\\ \;+\overset{\text{n}}{\underset{j=1}{\sum }}{\text{r}}_{ij}\left[g_{j}\left({\text{y}}_{j}\left(t-\pi \tau \right)\right)-g_{j}\left(\varpi x_{j}\left(t-\pi \tau \right)\right)\right] \end{array}$$

$$\begin{array}{l} \leq -\overset{\text{n}}{\underset{i=1}{\sum }}{\text{a}}_{i}\left|{\text{S}}_{{\text{E}}_{i}}\left(t\right)\right|+\overset{\text{n}}{\underset{i=1}{\sum }}\left[\;\overset{\text{n}}{\underset{j=1}{\sum }}\left[\overset{\text{u}}{\underset{\text{k}=1}{\sum }}\left|\left(M_{1_{j\text{k}}}\right)\left(M_{21_{\text{k}i}}\right)\right|\right]\left|{\text{S}}_{{\text{E}}_{j}}\left(t\right)\right|\right]\\ \;+\overset{\text{n}}{\underset{i=1}{\sum }}\left[\;\overset{\text{n}}{\underset{j=1}{\sum }}\left[\overset{\text{u}}{\underset{\text{k}=1}{\sum }}\left|\left(M_{1_{j\text{k}}}\right)\left(M_{3_{\text{k}i}}\right)\right|\left|{\text{S}}_{{\text{E}}_{j}}\left(t-\pi \tau \right)\right|\right]\right]\;\\ \;+\overset{\text{n}}{\underset{i=1}{\sum }}\left[\overset{\text{n}}{\underset{j=1}{\sum }}\left|{\text{q}}_{ij}\right|{\text{u}}_{j}\left|\left({\text{y}}_{j}\left(t\right)\right)-\left(\varpi x_{j}\left(t\right)\right)\right|\right]\\ \;+\overset{\text{n}}{\underset{i=1}{\sum }}\left[\overset{\text{n}}{\underset{j=1}{\sum }}\left|{\text{r}}_{ij}\right|{\text{k}}_{j}\left|\left({\text{y}}_{j}\left(t-\tau_{i}\right)\right)-\left(\varpi x_{j}\left(t-\pi \tau \right)\right)\right|\right] \end{array}$$

$$\begin{array}{l} =-\overset{\text{n}}{\underset{i=1}{\sum }}{\text{a}}_{i}\left|{\text{S}}_{{\text{E}}_{i}}\left(t\right)\right|+\overset{\text{n}}{\underset{i=1}{\sum }}\left[\;\overset{\text{n}}{\underset{j=1}{\sum }}\left[\overset{\text{u}}{\underset{\text{k}=1}{\sum }}\left|\left(M_{1_{j\text{k}}}\right)\left(M_{2_{\text{k}i}}\right)\right|\right]\right]\left|{\text{S}}_{{\text{E}}_{i}}\left(t\right)\right|\\ \;+\overset{\text{n}}{\underset{i=1}{\sum }}\left[\;\overset{\text{n}}{\underset{j=1}{\sum }}\left[\overset{\text{u}}{\underset{\text{k}=1}{\sum }}\left|\left(M_{1_{j\text{k}}}\right)\left(M_{3_{\text{k}i}}\right)\right|\right]\right]\left|{\text{S}}_{{\text{E}}_{j}}\left(t-\pi \tau \right)\right|\;\\ \;+\overset{\text{n}}{\underset{i=1}{\sum }}\left[\overset{\text{n}}{\underset{j=1}{\sum }}\left|{\text{q}}_{ij}\right|{\text{u}}_{j}\left|{\text{S}}_{{\text{E}}_{i}}\left(t\right)\right|\right]\;+\overset{\text{n}}{\underset{i=1}{\sum }}\left[\overset{\text{n}}{\underset{j=1}{\sum }}\left|{\text{r}}_{ij}\right|{\text{k}}_{j}\left|{\text{S}}_{{\text{E}}_{j}}\left(t-\pi \tau \right)\right|\right] \end{array}$$

$$\begin{array}{l} =-\overset{\text{n}}{\underset{i=1}{\sum }}\left[{\text{a}}_{\text{i}}-\left[\;\overset{\text{n}}{\underset{j=1}{\sum }}\left[\overset{\text{u}}{\underset{\text{k}=1}{\sum }}\left|\left(M_{1_{j\text{k}}}\right)\left(M_{2_{\text{k}i}}\right)\right|\right]\right]\;+\left[\overset{\text{n}}{\underset{j=1}{\sum }}\left|{\text{q}}_{ij}\right|{\text{u}}_{j}\right]\right]\left|{\text{S}}_{{\text{E}}_{i}}\left(\text{t}\right)\right|\\ \;\;\;\;+\left[\overset{\text{n}}{\underset{i=1}{\sum }}\left[\;\overset{\text{n}}{\underset{j=1}{\sum }}\left[\overset{\text{u}}{\underset{\text{k}=1}{\sum }}\left|\left(M_{1_{j\text{k}}}\right)\left(M_{3_{\text{k}i}}\right)\right|\right]\right]+\overset{\text{n}}{\underset{i=1}{\sum }}\left[\overset{\text{n}}{\underset{j=1}{\sum }}\left|{\text{r}}_{ij}\right|{\text{k}}_{j}\right]\right]\left|{\text{S}}_{{\text{E}}_{i}}\left(t-\pi \tau \right)\right| \end{array}$$

$$\begin{array}{l} \leq \underset{1\leq i\leq \text{n}}{\text{max}}\left[\overset{\text{n}}{\underset{j=1}{\sum }}\left[\overset{\text{u}}{\underset{\text{k}=1}{\sum }}\left|\left(M_{1_{j\text{k}}}\right)\left(M_{3_{\text{k}i}}\right)\right|\right]\;+\overset{\text{n}}{\underset{j=1}{\sum }}\left|{\text{r}}_{ji}\right|{\text{k}}_{i}\;\right]\overset{\text{n}}{\underset{i=1}{\sum }}\left|{\text{S}}_{{\text{E}}_{i}}\left(t-\pi \tau \right)\right|\\ \;-\underset{1\leq i\leq \text{n}}{\text{min}}\left[{\text{a}}_{i}-\overset{\text{n}}{\underset{j=1}{\sum }}\left[\overset{\text{u}}{\underset{\text{k}=1}{\sum }}\left|\left(M_{1_{j\text{k}}}\right)\left(M_{2_{\text{k}i}}\right)\right|\right]-\overset{\text{n}}{\underset{j=1}{\sum }}\left|{\text{q}}_{ji}\right|{\text{u}}_{i}\right]\overset{\text{n}}{\underset{i=1}{\sum }}\left|\left|{\text{S}}_{{\text{E}}_{i}}\left(t\right)\right|\right| \end{array}$$

$$\leq -\Psi_{1}V\left({\text{S}}_{{\text{E}}_{i}}\left(t\right)\right)+\Psi_{2}\underset{t-\tau \leq \text{s}\leq t}{\text{sup}}V\left({\text{S}}_{{\text{E}}_{i}}\left(\text{s}\right)\right)$$ (18)

It should be noted that

$$\underset{t-\tau \leq \text{s}\leq \text{t}}{\text{sup}}V\;\left({\text{S}}_{{\text{E}}_{i}}\left(\text{s}\right)\right)\leq V\;\left({\text{S}}_{{\text{E}}_{i}}\left(t\right)\right)$$ (19)

Then, based on (18), (19) and Theorem 10, assuming a constant C greater than zero, one has

$$\left\{ \begin{array}{c} D_{+}^{\alpha }\;V\left({\text{S}}_{{\text{E}}_{i}}\left(t\right)\right)\leq -\left(\Psi_{1}-\Psi_{2}\right)V\left({\text{S}}_{{\text{E}}_{i}}\left(t\right)\right)\\ \Psi_{1}-\Psi_{2}\geq \mathbb{C} \end{array}\right.$$ (20)

The following is how we construct Eq (20):

$$D_{+}^{\alpha }\;V\left({\text{S}}_{{\text{E}}_{i}}\left(t\right)\right)\leq \mathbb{C}V\left({\text{S}}_{{\text{E}}_{i}}\left(t\right)\right)$$ (21)

So,

$$\left|\right|{\text{S}}_{{\text{E}}_{i}}\left(t\right)||=||\left({\text{y}}_{j}\;\left(t\right)\right)-\left(\varpi x_{j}\left(t\right)\right)||$$

$$=\overset{\text{n}}{\underset{i=1}{\sum }}||\left({\text{y}}_{j}\;\left(t\right)\right)-\left(\varpi x_{j}\left(t\right)\right)\;||$$ (22)

Finally, the Eq (14) is stable as $\left\|{S_{E}}_{i}\left(t\right)\right\|\to \;0$ as t approaches infinity. This thoroughly demonstrates the proof. ■

**Remark 1**. When the value of ϖ equals 1 and (H) holds, the synchronization multi-FONNSMD is called the "complete synchronization".

**Remark 2**. When the value of ϖ equals −1 and (H) holds, the synchronization of multi-FONNSMD is referred to as the "anti-synchronization".

## 4. Symmetry encryption

This section will perform the encryption and decryption of a message. Symmetric cryptographic algorithms (SCA) and the public key are first used as an original message that is called plaintext (PTEXT) to generate the ciphertext (CTEXT), and the CTEXT is re-encrypted to achieve double encryption using synchronization. It consists of the encrypter (RSS and encryption function of SCA) and the decrypter (DSS and decryption function of SCAL). We use (1) as DSS and (2) as RSS, and we have shown that the Eq (14) is $\left\|{S_{E}}_{i}\left(t\right)\right\|\to \;0$, the synchronization is achieved. The sender uses multi-FONNSMD (1), and the receiver uses multi-FONNSMD (2), and they both choose $x_{3}\left(t\right)$ and $y_{3}\left(t\right)$ as the public keys after a time, which means that the two FONNSMDs synchronised. The sender picks up the data from $x_{3}\left(t\right)$ to obtain the secret keys for encryption while the receiver picks up data from $y_{3}\left(t\right)$ to obtain secret keys (SKEY) and decrypt the CTEXT.

PTEXT and CTEXT are represented in numbers; thus, every letter from the alphabet is substituted by the appropriate numeral associated with ASCII representation. The formula for SCA encryption and decryption is given by:

$$\begin{array}{l} C_{\text{T}}{}_{i}={\text{T}}_{\text{T}}{}_{i}+K_{i}\;\left(\text{mod}\;38\right)\\ {\text{T}}_{\text{T}}{}_{i}=C_{\text{T}}{}_{i}-K_{i}\;\left(\text{mod}\;38\right) \end{array}$$ (23)

With $K_{i}$ The SKEYs are used to mask the message. These SKEY are completely applied once. The sender can randomly adjust the initial conditions of multi-FONNSMD (1) for the next time. This is because chaotic systems are sensitive to the initial state.

## 5. Proposed NNSE algorithm

**Fig 1:**
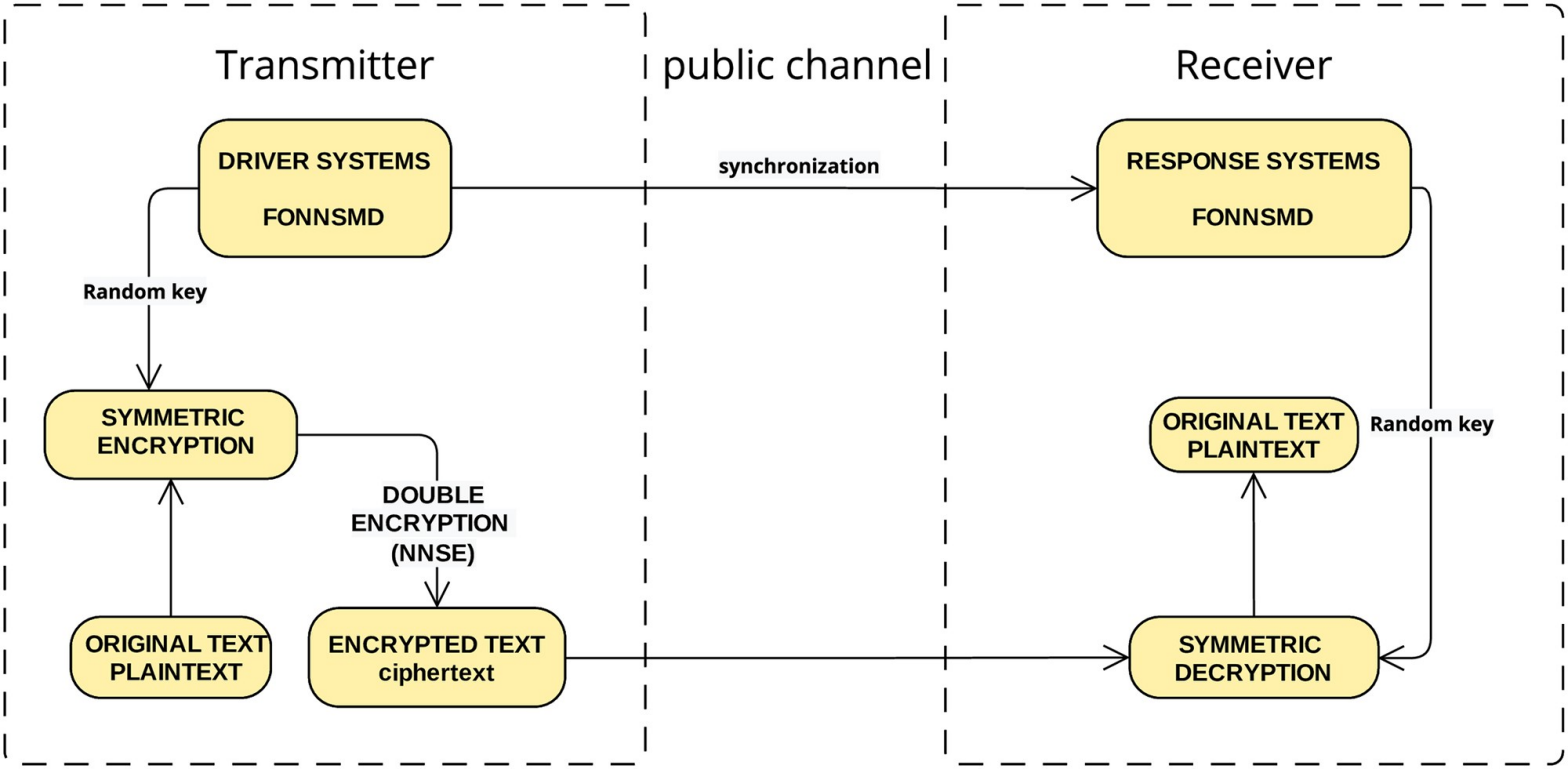
Encryption flowchart.

**Problem**: How to enforce double encryption of two multi-FONNSMD with different initial conditions using the SCA encryption/decryption schemes and chaotic synchronization. We can overcome this problem using the following steps: Chaotic Synchronization + SCA = NNSE. We can summarize this algorithm as shown in Fig 1.

You may summarize the entire design technique as follows:

**Step 1**: Construct the multi-FONNSMD of the DSS (1) and the RSS (2), respectively.
**Step 2**: Define the EDSMC can then be obtained using MATLAB simulation.
**Step 3**: A message is converted from PTEXT by an ASCII encoder.
Input PTEXT and the PKEY to form a CTEXT by SCA encryption.
**Step 4**: Both sender and receiver agree that private keys (PKEY) are from the values of integers part of 10000x and 10000y.
**Step 5**: The sender picks up the data $x_{3}\left(t\right)$ to obtain the secret keys for encryption while the receiver picks up data y_3_(t) to obtain SKEY and decrypts the CTEXT.
**Step 6**: Since the multi-FONNSMD is with the time-delay, they both decide to change the values of τ_1_, τ_2_ and τ_3_ after every five units of a message to maintain security. They increase the values of τ_1_, τ_2_ and τ_3_ by 0.1.
**Step 7**: The formula for encryption is $C_{i}=P_{i}+K_{i}\;(mod\;38)$, and decryption is $P_{i}=C_{i}-K_{i}\;(mod\;38)$
**Step 8**: Enter PTEXT and the PKEY to construct a CTEXT using the SCA encryption.
**Step 9**: The CTEXT is then forwarded to the DSS to get the encrypted signal.
**Step 10**: The PTEXT is obtained using PKEY and the SCA decryption scheme.
**Step 11**: We can retrieve the CTEXT from the RSS based on the encrypted signal.
**Step 12**: The plaintext is recovered via the SCA decryption scheme and the PKEY.

## 6. Results, numerical verification and discussion

In this section, the suggested definition with three numerical examples is disclosed to validate the feasibility and effectiveness of the achieved results by using multi-FONNSMD systems as the DRS. We conducted three experiments to see the difference between non-identical synchronization of multi-FONNSMD with and without multi-time delay. The parameters are selected as α_i_ = [1, 0.99, 0.98, 0.97, 0.96, 0.95], *τ*_1_ = 0.5, *τ*_2_ = 1.5, *τ*_3_ = 2.5, *U* = (*I*_1_ *I*_2_ *I*_3_))^*T*^ = *V* = (0 0 0)^*T*^ and *f*(.) = tanh(.), where i = 1,2,3

The initial values of x(t) and *y*(t) are taken as:

$$x_{\text{i}}\left(0\right)=-0.22$$

$${\text{y}}_{\text{i}}\left(0\right)=-0.22$$

where i = 1,2,3

**Example 1**: Non-identical synchronization of multi-FONNSMD with multi-time delay

$$A=P=diag\left(\begin{array}{ccc} 1 & 1 & 1 \end{array}\right)$$

$$B=\left[\begin{array}{lll} 0.2 & 0.5 & 5.5\\ 0.5 & 0.5 & 5.1\\ -0.5 & -0.1 & -0.5 \end{array}\right]$$

$$C=\left[\begin{array}{ccc} 0.7 & 0.7 & 4.1\\ 0.1 & 0.1 & 2.5\\ -50.1 & -100.1 & 2.5 \end{array}\right]$$

$$Q=\left[\begin{array}{ccc} 0.2 & 0.3 & 7.5\\ 0.5 & 0.5 & 7.5\\ -0.5 & -3 & -0.5 \end{array}\right]$$

$$R=\left[\begin{array}{ccc} 0.3 & 5.5 & 5.5\\ 0.5 & 2.5 & 5.5\\ 7 & -50.5 & -2.5 \end{array}\right]$$

**Example 2**: Non-identical synchronization of multi-FONNSMD with multi time-varying delay

$$A=diag\left(\begin{array}{ccc} 1 & 2 & 2 \end{array}\right)$$

$$P=diag\left(\begin{array}{ccc} 2 & 2 & 2 \end{array}\right)$$

$$B=\left[\begin{array}{lll} 0.2 & 0.5 & 5.5\\ 0.5 & 0.5 & 5.1\\ -0.5 & -0.1 & -0.5 \end{array}\right]$$

$$C=\left[\begin{array}{ccc} 0.7 & 0.7 & 4.1\\ 0.1 & 0.1 & 2.5\\ -50.1 & -100.1 & 2.5 \end{array}\right]$$

$$Q=\left[\begin{array}{ccc} 0.2 & 0.3 & 7.5\\ 0.5 & 0.5 & 7.5\\ -0.5 & -3 & -0.5 \end{array}\right]$$

$$R=\left[\begin{array}{ccc} 0.3 & 5.5 & 5.5\\ 0.5 & 2.5 & 5.5\\ -50.1 & -50.5 & -2.5 \end{array}\right]$$

**Example 3**: Non-identical synchronization of multi-FONNSMD without delay

$$A=diag\left(\begin{array}{ccc} 10.5 & 10.5 & 0.5 \end{array}\right)$$

$$P=diag\left(\begin{array}{ccc} 20.5 & 20.5 & 20.5 \end{array}\right)$$

$$B=\left[\begin{array}{lll} 0.2 & 0.5 & 5.5\\ 0.5 & 0.5 & 5.1\\ -0.5 & -0.1 & -0.5 \end{array}\right]$$

$$C=\left[\begin{array}{ccc} 0.7 & 0.7 & 4.1\\ 0.1 & 0.1 & 2.5\\ -50.1 & -100.1 & 2.5 \end{array}\right]$$

$$Q=\left[\begin{array}{ccc} 0.2 & 0.3 & 7.5\\ 0.5 & 0.5 & 7.5\\ -0.5 & -3 & -0.5 \end{array}\right]$$

$$R=\left[\begin{array}{ccc} 0.3 & 5.5 & 5.5\\ 0.5 & 2.5 & 5.5\\ -50.1 & -50.5 & -2.5 \end{array}\right]$$

After obtaining all the parameters for each experiment, we can answer the above experiments based on the NNSE algorithm steps.

**Step 1**: Established the multi-FONNSMD from DSS (1) and RSS (2) as:

$$D^{\alpha_{1}}x\left(t\right)=-\text{A}x\left(t\right)+\text{B}f\left(x\left(t\right)\right)+\overset{3}{\underset{\pi =1}{\sum }}\text{C}f\left(x\left(t-\pi \tau \right)\right)+U$$ (24)

And

$$D^{\alpha_{2}}\text{y}\left(t\right)=-\text{Py}\left(t\right)+\text{Q}g\left(\text{y}\left(t\right)\right)+\overset{3}{\underset{\pi =1}{\sum }}Rg\left(\text{y}\left(t-\pi \tau \right)\right)+\text{V}+{\text{S}}_{\text{C}}\left(t\right)$$ (25)

**Fig 2:**
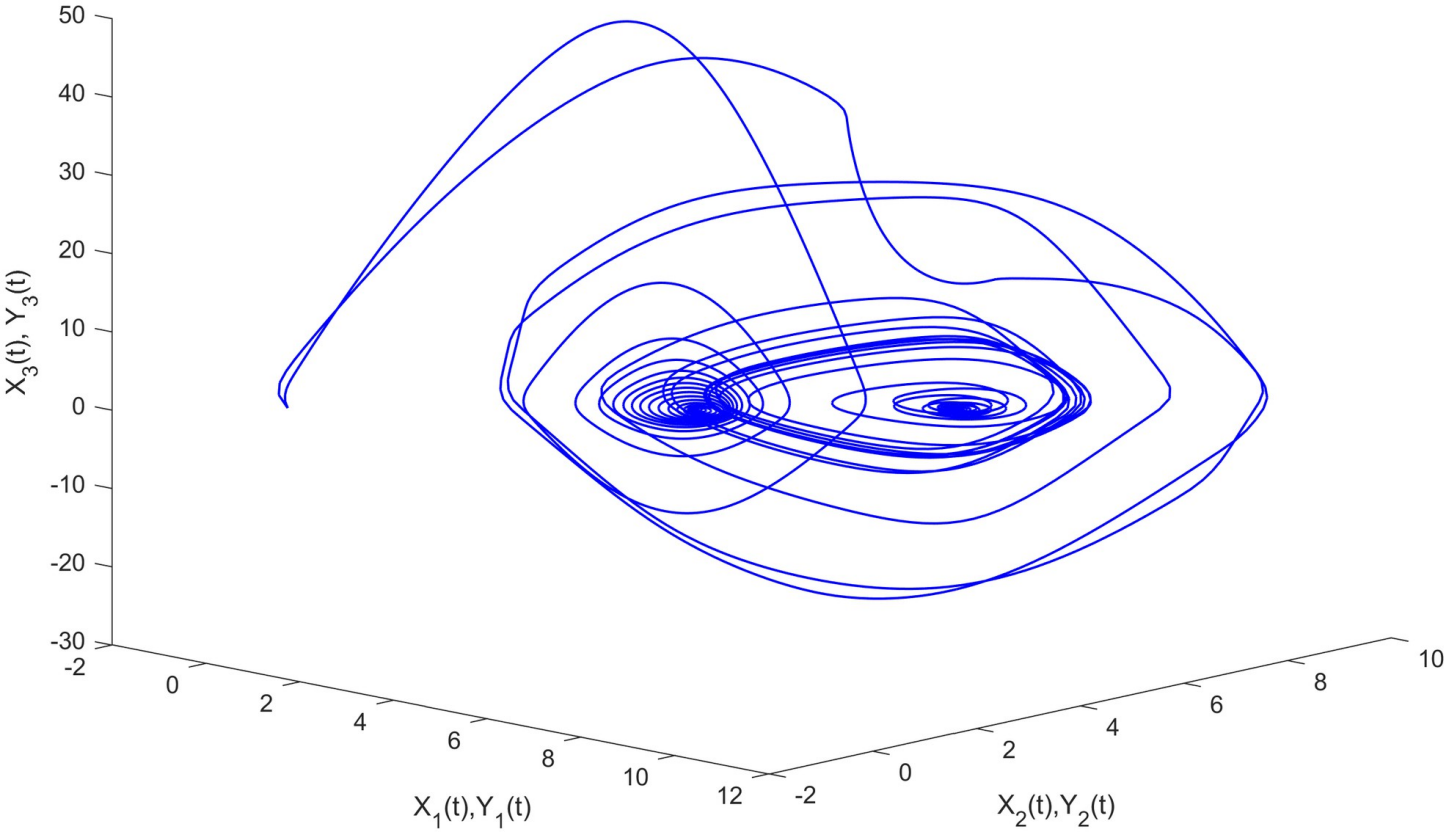
The chaotic attractor of multi-FONNSMD systems (24) and (25)—Phase portrait.

A graphical illustration of the phase portrait of the Eqs (24) and (25) is shown in Fig 2.

**Fig 3:**
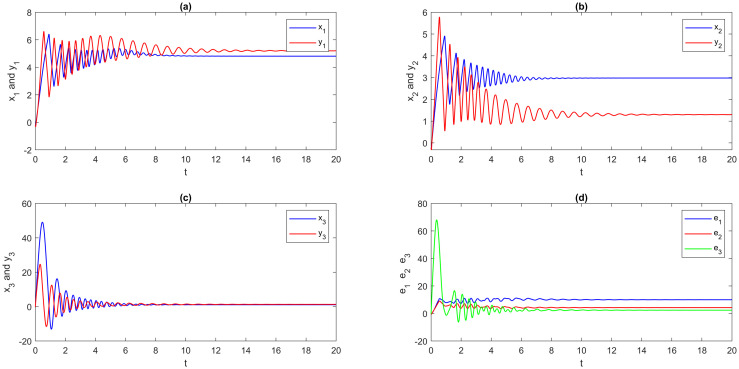
Multi-time delay of multi-FONNSMD synchronization without controller activation. (a) x_1_ versus y_1_, (b) x_2_ versus y_2_, (c) x_3_ versus y_3_, and (d) Synchronization error.

**Fig 4:**
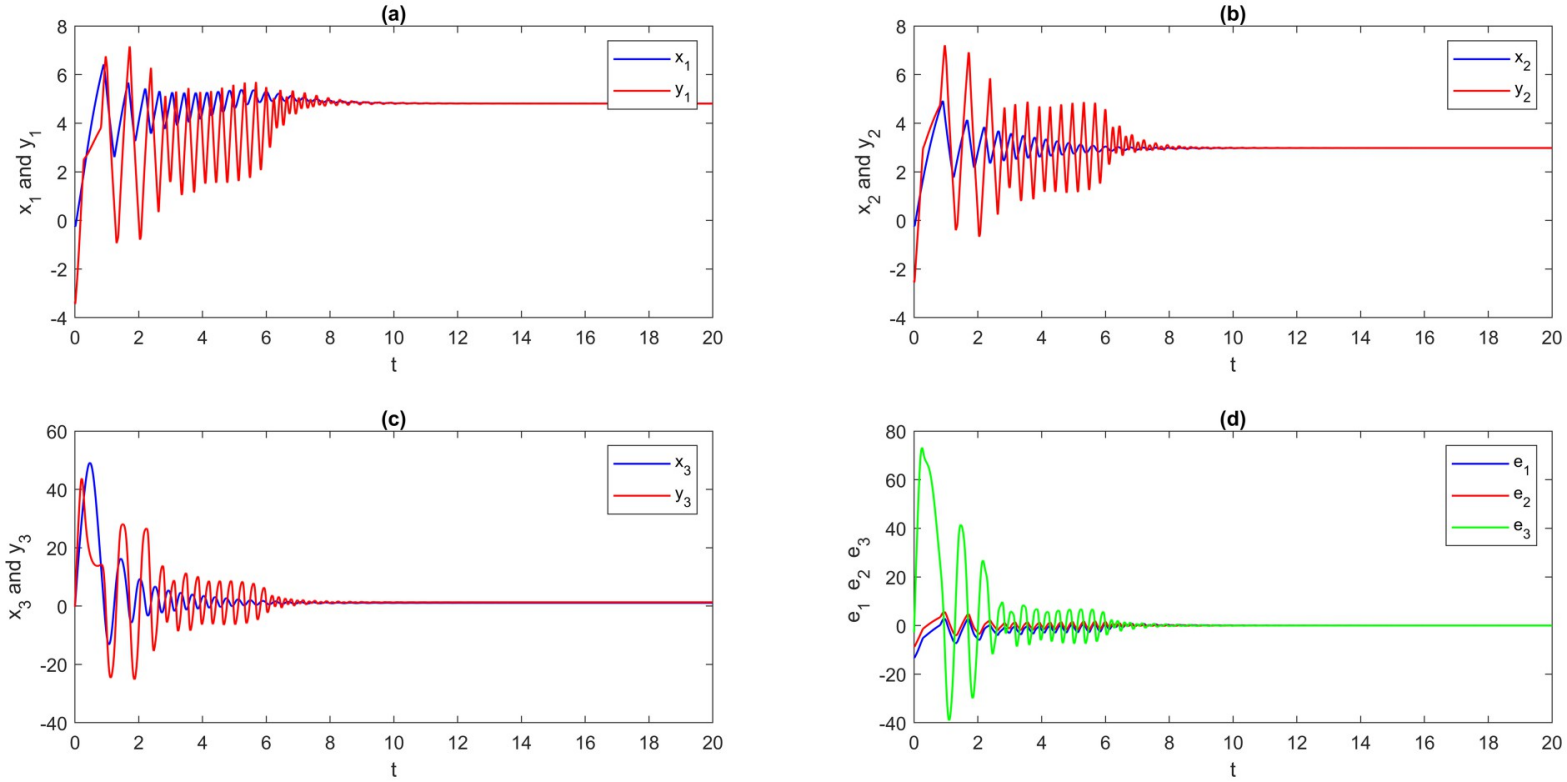
Multi-time delay of multi-FONNSMD synchronization with controller activation. (a) x_1_ versus y_1_, (b) x_2_ versus y_2_, (c) x_3_ versus y_3_, and (d) Synchronization error.

**Step 2**: The EDSMC is obtained using MATLAB simulation. With the initial values and suggested network parameters above, we answer the numerical solutions of the DSS (24), RSS (25), and error system (14). The numerical simulation method is presented here for solving fractional differential equations by using the step-by-step iterative method and MATLAB tools. The graphical analysis displays the findings using this program. A visual representation is provided in Fig 3, which portrays the outcomes of multi-FONNSMD synchronization without controller activation, while controller activation is shown in Fig 4. It is seen in Fig 3(d) that the error state does not converge to zero because the control has not been enabled, and it can also be seen in Fig 4(d) that when the controller is enabled, the convergence of error state in a finite period is demonstrated.

**Fig 5:**
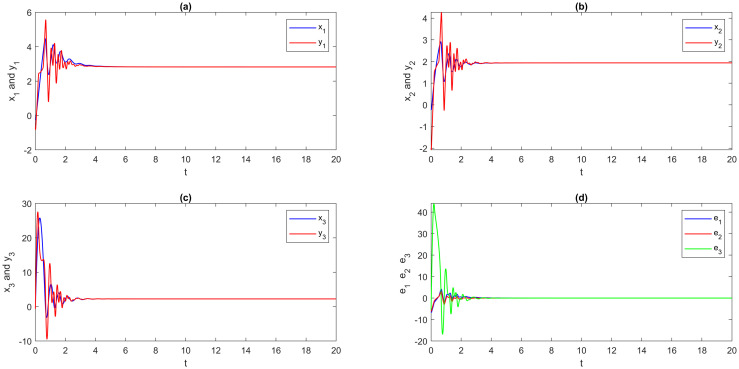
Multi time-varying delay of multi-FONNSMD synchronization with controller activation. (a) x_1_ versus y_1_, (b) x_2_ versus y_2_, (c) x_3_ versus y_3_, and (d) Synchronization error.

**Fig 6:**
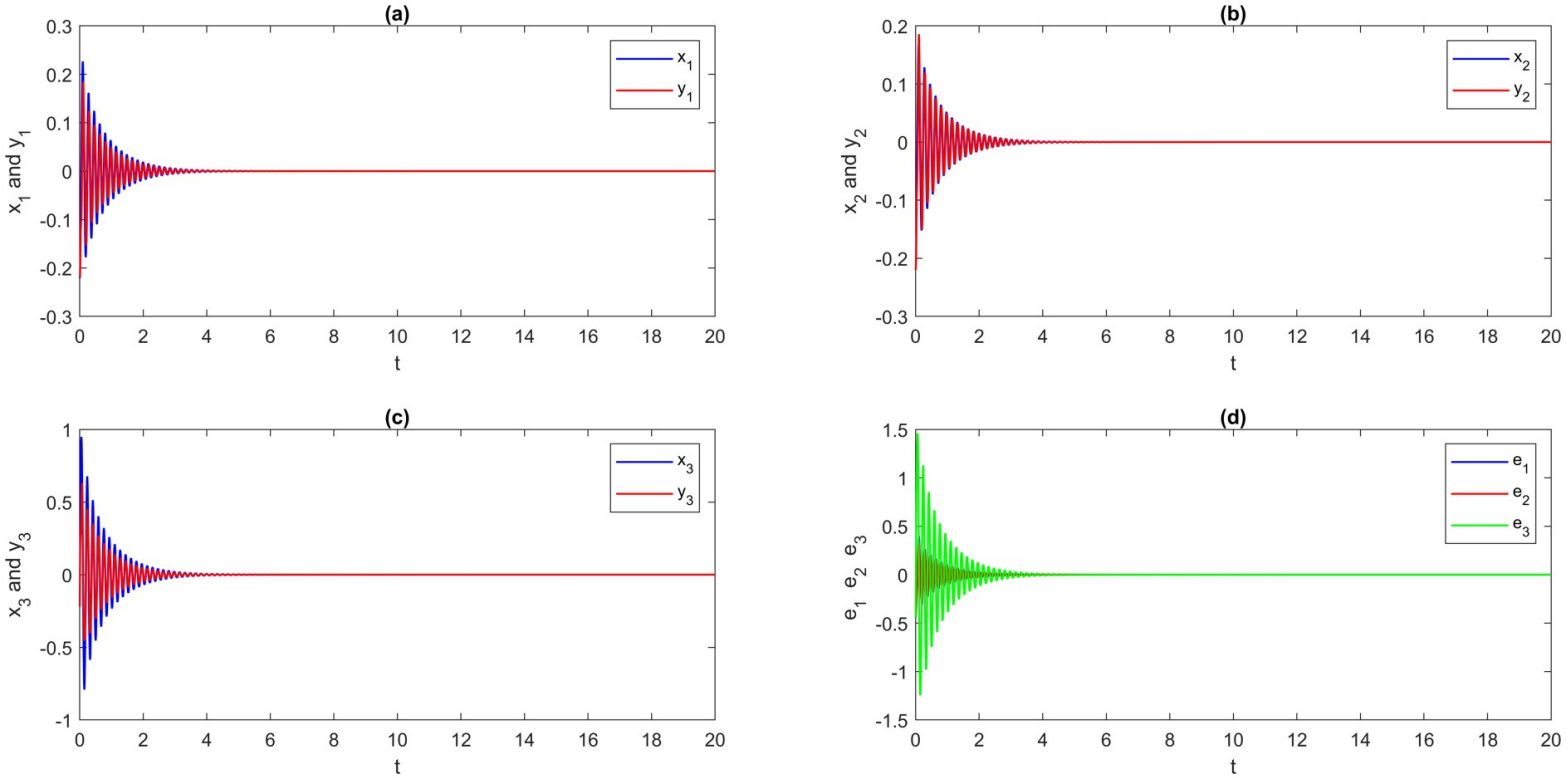
Without delay, multi-FONNSMD synchronization with controller activation. (a) x_1_ versus y_1_, (b) x_2_ versus y_2_, (c) x_3_ versus y_3_, and (d) Synchronization error.

From Fig 4, it is easy to see that the DRS (24) and (25) are globally Mittag-Leffler asymptotic projective synchronization, which validates the reasonableness and usefulness of the conditions given in the definition 5. To further confirm that the DRS (24) and (25) are globally Mittag-Leffler asymptotic projective synchronization, we indicate a multi-time-varying delay without time-delay for simulation. Moreover, the results are shown in Figs 5 and 6; the order of fractional-order and the initial value will not change the global Mittag-Leffler synchronization of DRS (24) and (25) with different values of parameters.

Based on Examples 1–3, Table 2 encloses various results of dynamic synchronization error values for different values of parameters of multi fractional-order in the system (24) and system (25). At this point, experiments are conducted with multi-time delay, multi time-varying delays and without delay. As seen in Table 2, as time increases, the dynamic synchronization errors of ${S_{E}\left(t\right)}_{1},\;S_{E}{\left(t\right)}_{2}$, and $S_{E}{\left(t\right)}_{3}$ converge to zero. All the examples of dynamic errors show the same performance. Initially, the error value reduces, and after some time, it increases subsequently and eventually decreases throughout the time. In conclusion, Table 3 and S1 and S2 Tables are shown that the whole time taken for dynamic synchronization errors converges to zero.

**Table 2:** Result for dynamic synchronization errors for the multi-time delay, multi time-varying delay and without delay.

t	Example 1: Multi-time delay			Example 2: Multi time-varying delay			Example 3: Without delay		
	${S_{E}}_{1}\left(t\right)$	$S_{E_{2}}\left(t\right)$	${S_{E}}_{3}\left(t\right)$	${S_{E}}_{1}\left(t\right)$	$S_{E_{2}}\left(t\right)$	${S_{E}}_{3}\left(t\right)$	${S_{E}}_{1}\left(t\right)$	$S_{E_{2}}\left(t\right)$	${S_{E}}_{3}\left(t\right)$
0.2	-7.635074	-3.555392	69.219102	-2.245052	-1.520800	43.668359	-0.309077	-0.283690	0.173145
0.4	-3.851019	-0.082654	66.828019	-0.282647	0.064876	31.754649	-0.132708	-0.107217	0.705430
0.6	-1.768717	1.763905	54.270791	2.729043	2.082869	14.694244	0.053379	0.060519	0.624069
0.8	0.027315	3.283361	27.860997	-0.150939	-1.126820	-14.830215	0.131721	0.121321	0.129667
1.0	2.002976	4.702250	-21.822156	0.627415	-0.116200	12.685434	0.075061	0.061704	-0.281366
1.2	-5.416797	-2.078373	-25.805573	1.663847	0.497237	1.471384	-0.024654	-0.028759	-0.296369
1.4	-5.274272	-2.445515	36.517509	-0.704473	-1.639829	0.344665	-0.065200	-0.060144	-0.042823
1.6	0.820877	2.925084	29.578378	0.696254	-0.270170	1.597438	-0.031423	-0.025457	0.155458
1.8	-0.656675	1.681916	-27.569091	0.865260	-0.076485	-3.991734	0.018813	0.019647	0.137339
2.0	-5.539274	-2.956726	-1.465992	0.415925	-0.278293	-0.657083	0.033062	0.029699	-0.001114

**Table 3:** Time for error dynamics to converge to zero for the multi-FONNSMD system with multi-time delay.

t	Multi-time delay		
	${S_{E}}_{1}\left(t\right)$	$S_{E_{2}}\left(t\right)$	${S_{E}}_{3}\left(t\right)$
0.5	-2.783312	0.881577	62.943161
1.0	2.0029766	4.702250	-21.822156
1.5	-2.050509	0.390226	40.653769
2.0	-5.539274	-2.956726	-1.465992
2.5	-2.230595	-0.107194	-9.765992
3.0	-2.966520	-1.175349	4.761501
3.5	-0.649199	0.884686	7.212369
4.0	-1.765203	-0.226732	-5.061299
4.5	-1.231654	-0.144178	6.081291
5.0	-0.280444	0.821304	-6.803661
5.5	-2.701274	-1.7190548	2.582611
6.0	1.175210	1.646657	-0.481021
6.5	-0.271424	-0.028015	-2.016353
7.0	0.246905	0.132753	0.592116
7.5	0.097735	-0.055733	0.679700
8.0	0.191682	0.021054	-0.325423
8.5	0.091803	0.004132	-0.216673
9.0	0.064219	-0.000897	0.199014
9.5	0.067831	0.006754	0.031487
10.0	0.013169	-0.010749	-0.081708
10.5	0.012888	-0.003985	0.027590
11.0	0.028697	0.006889	0.018931
11.5	0.006457	-0.002450	-0.017540
12.0	0.000793	-0.003668	0.007673
12.5	0.009605	0.001986	0.005627
13.0	0.004212	-0.000047	-0.005399
13.5	0.000623	-0.001451	0.003374
14.0	0.003275	0.000272	0.002938
14.5	0.002031	-0.000027	-0.001703
15.0	0.000571	-0.000546	0.001188
15.5	0.001212	-0.000034	0.001570
16.0	0.000765	-0.000076	-0.000270
16.5	0.000113	-0.000246	0.000459
17.0	0.000191	-0.000063	0.000728
17.5	-0.000047	-0.000046	6.268655
18.0	-0.000404	-0.000097	0.000194
18.5	-0.000497	-0.000021	0.000285
19.0	-0.000669	0.000008	0.000030
19.5	-0.000897	0.000005	0.000019
20.0	-0.001032	0.000044	0.000028

**Fig 7:**
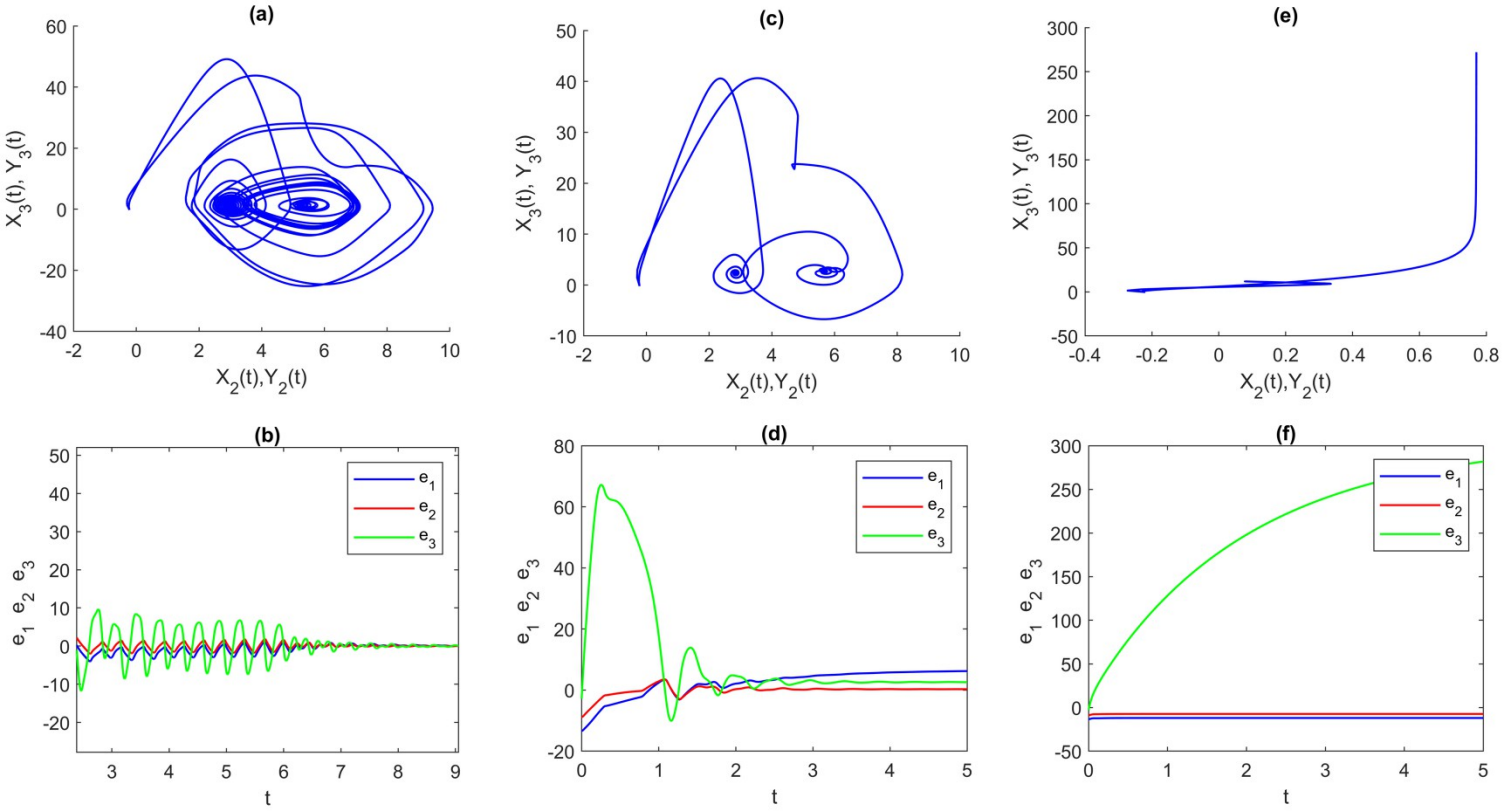
Multi-time delay of multi-FONNSMD synchronization with different value of parameters. (a) A = P = diag[1 1 1], (c) A = diag[1 2 2], P = diag[2 2 2], and (e) A = diag[10.5 10.5 0.5], P = diag[20.5 20.5 20.5]. (a), (c) and (e) are synchronization of FONNSMD systems while (b), (d) and (f) are dynamic error of FONNSMD systems.

**Fig 8:**
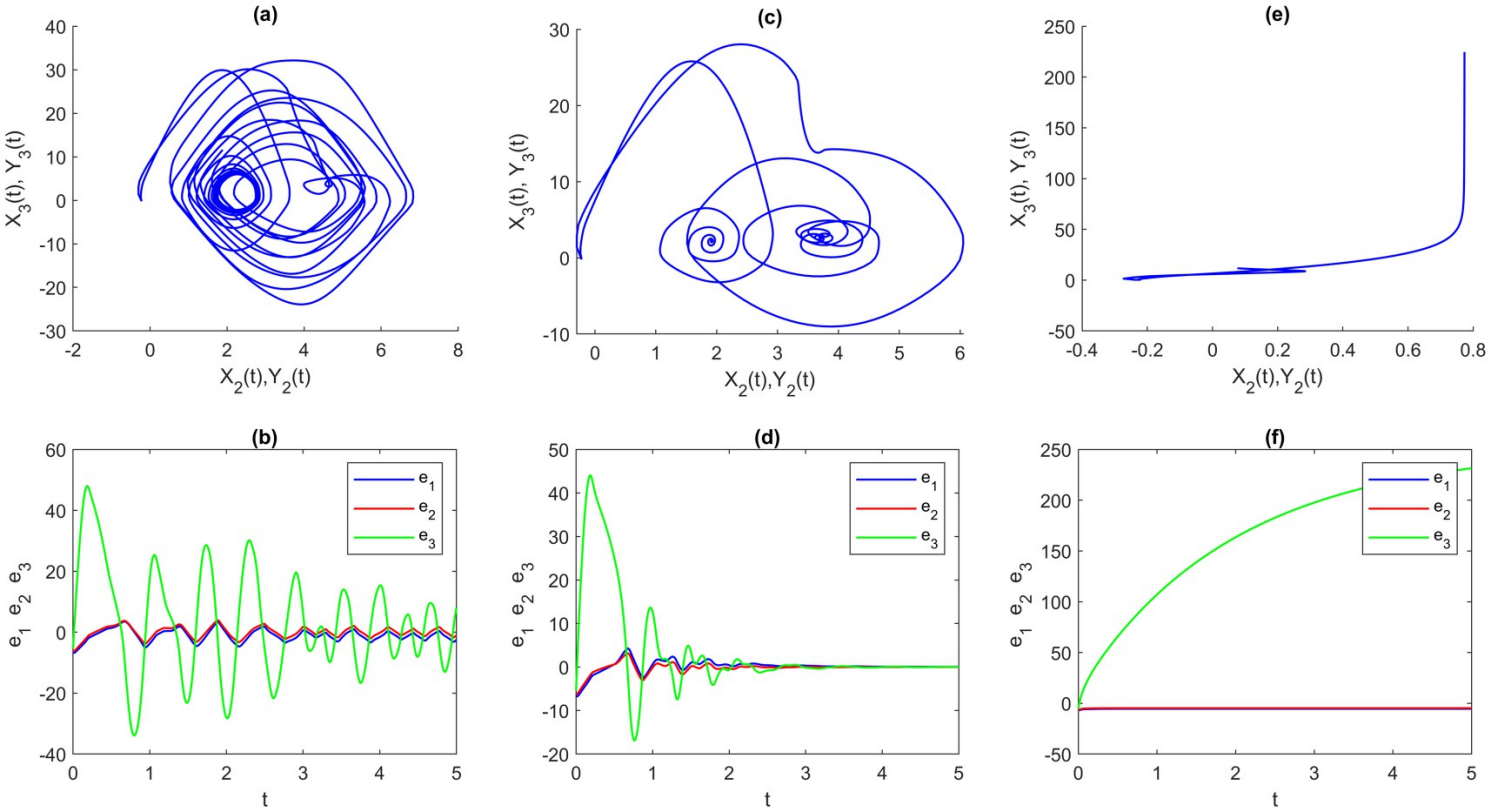
Multi time-varying delay of multi-FONNSMD synchronization with different value of parameters. (a) A = P = diag[1 1 1], (b) A = diag[1 2 2], P = diag[2 2 2], and (c) A = diag[10.5 10.5 0.5], P = diag[20.5 20.5 20.5]. (a), (c) and (e) are synchronization of FONNSMD systems while (b), (d) and (f) are dynamic error of FONNSMD systems.

**Fig 9:**
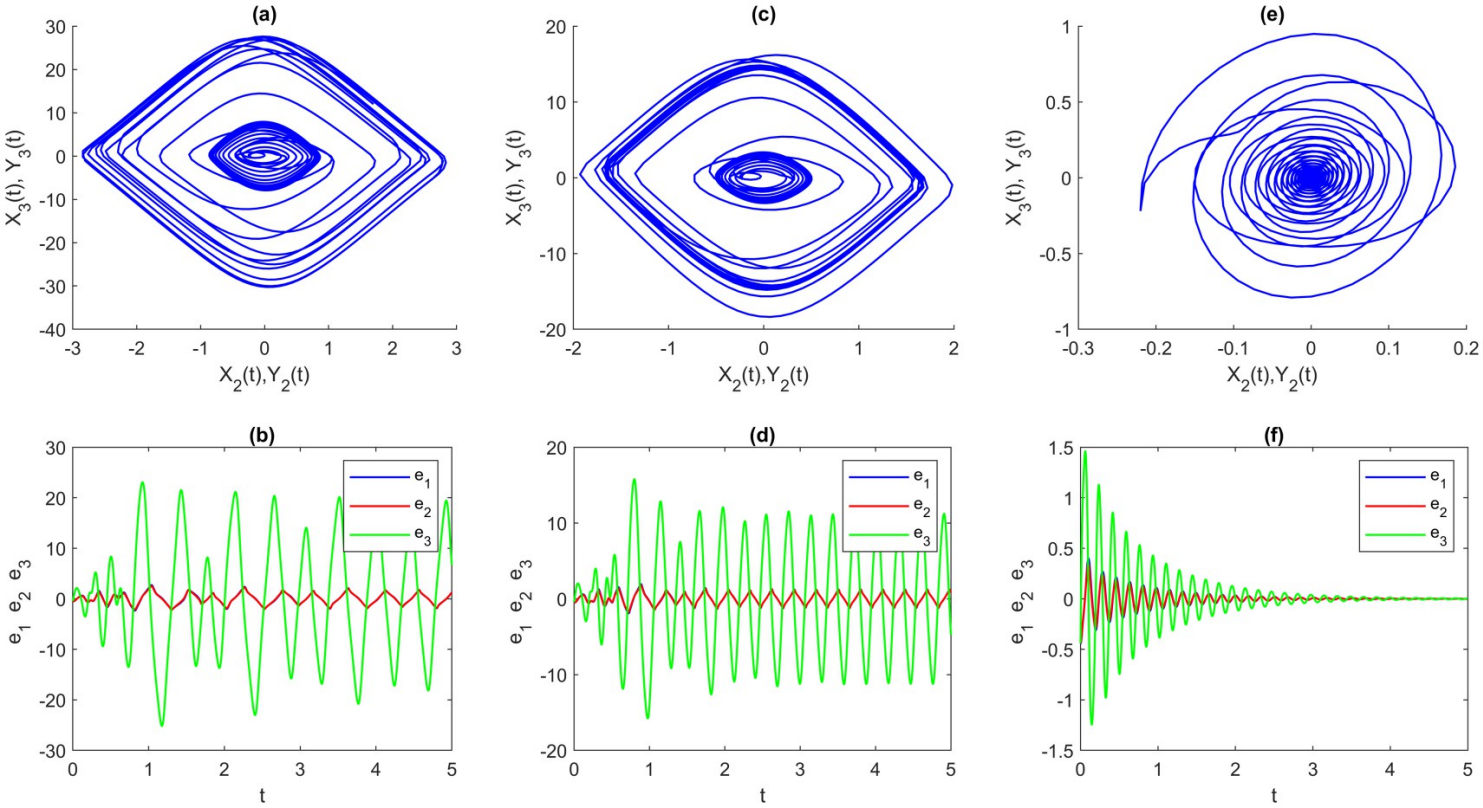
Without delay multi-FONNSMD synchronization with different value of parameters. (a) A = P = diag[1 1 1], (b) A = diag[1 2 2], P = diag[2 2 2], and (c) A = diag[10.5 10.5 0.5], P = diag[20.5 20.5 20.5]. (a), (c) and (e) are synchronization of FONNSMD systems while (b), (d) and (f) are dynamic error of FONNSMD systems.

Figs 7–9 show another experiment was done to see the effect of different parameters on all three types of multi-FONNSMD with the same multi fractional-order, which we called non-identical synchronization. We can see that only Figs 7(a), 8(b) and 9(c), the multi-FONNSMD system are chaotic and synchronized, while the others are not synchronized. From Figs 7–9, we can conclude the results in Table 4, where the chaotic systems appear for a wide range of system parameters but in a different range of multi fractional-order.

**Table 4:** The multi-FONNSMD system performance with different values of a parameter.

	A = P = diag[1 1 1]	A = diag[1 2 2] P = diag[2 2 2]	A = diag[10.5 10.5 0.5] P = diag[20.5 20.5 20.5]
Multi-time delay	chaotic	No synchronization	No chaotic
Multi time-varying delay	No synchronization	chaotic	No chaotic
Without delay	No synchronization	No synchronization	chaotic

Here, we choose example one for the next step.

**Step 3**: A message is converted from PTEXT by an ASCII encoder, and the space between two words is assigned by 10. The result is shown in Table 5.

**Table 5:** ASCII value.

Unit message	N	E	U	R	A	L	-	N	E	T	W	O	R	K	S
ASCII	78	69	85	82	65	76	10	78	69	84	87	79	82	75	83

Plaintext: NEURAL NETWORKS

**Step 4**: Both sender and receiver agree that private keys (PKEY) are from the values of integers that are part of 1000000x and 1000000y.

**Step 5**: The sender picks up the data from $x_{3}\;(t)$ to obtain the secret keys for encryption while the receiver picks up the data from y_3_(t) to get the SKEY and decrypt the CTEXT.

**Fig 10:**
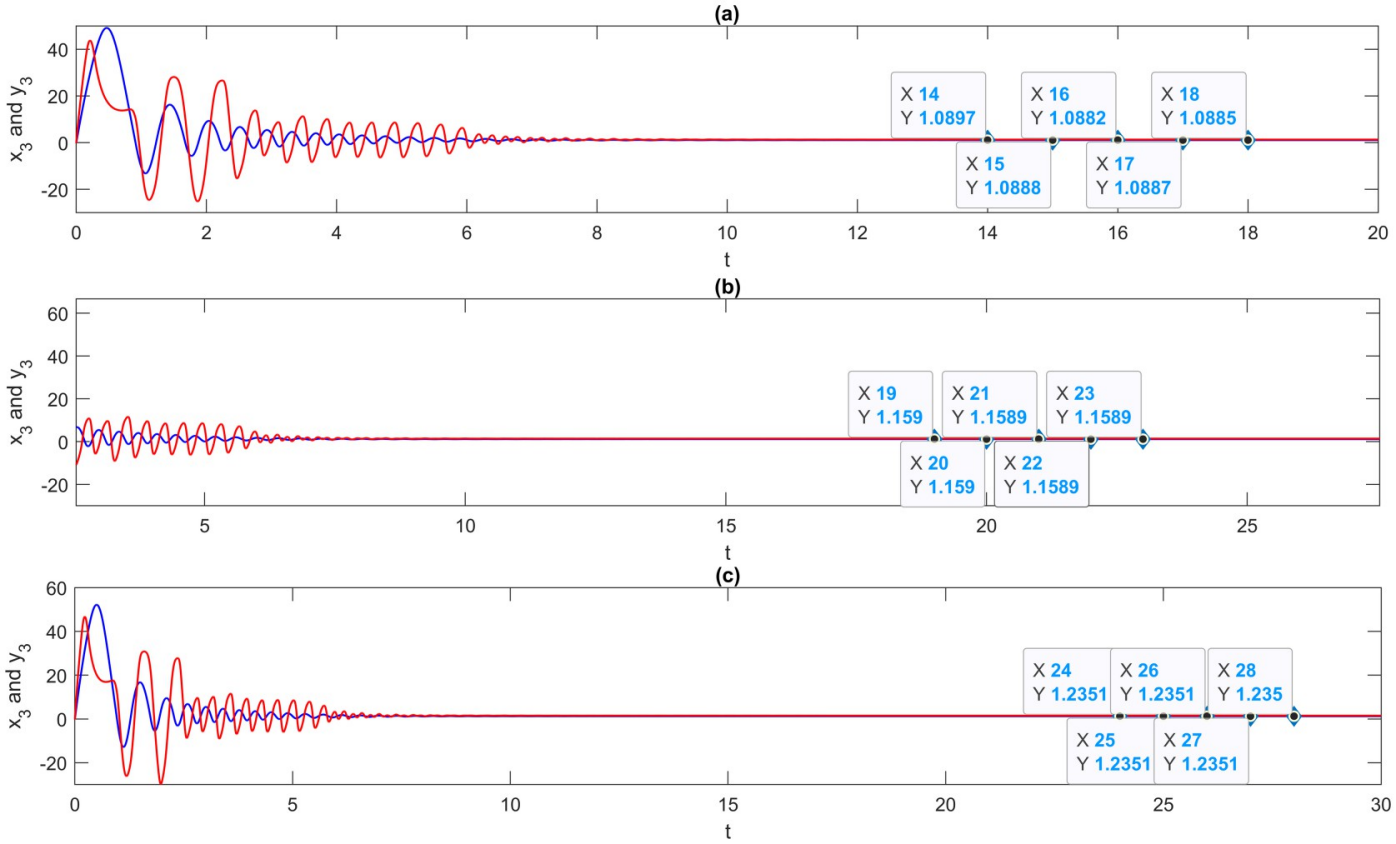
Phase portrait of $x_{3}(t)$ and y_3_(*t*) of multi-FONNSMD synchronization with multi-time delay [τ_1_, τ_2_, τ_3_]. (a) [1.5,2.5,3.5], (b) [1.6,2.6,3.6], and (c) [1.7,2.7,3.7].

**Step 6**: Since the multi-FONNSMD is with the multi-time delay, they agree to alternate values of τ_1_, τ_2_ and τ_3_ after every five units of a message. They increase the values of τ_1_, τ_2_ and τ_3_ by 0.1, as shown in Fig 10.

**Step 7**: The formula for encryption is $C_{i}=P_{i}+K_{i}\;(mod\;38)$, and decryption is $P_{i}=C_{i}-K_{i}\;(mod\;38)$

**Step 8**: Enter PTEXT and the PKEY to construct a CTEXT using the SCA encryption function. The result is shown in Table 6.

**Table 6:** Encryption of PTEXT to CTEXT.

Time *τ*_1_	Time *τ*_2_	Time *τ*_3_	Time *t*	*x*_3_(*t*)	Keys *k*	Plaintext *P*	Ciphertext ${C_{T}}_{i}={P_{T}}_{i}+K_{i}$ (mod 38),
1.5	2.5	3.5	14.0	1.089718	1089718	N (78)	32
			15.0	1.088824	1088824	E (69)	3
			16.0	1.088195	1088195	U (85)	36
			17.0	1.088692	1088692	R (82)	36
			18.0	1.088484	1088484	A (65)	1
1.6	2.6	3.6	19.0	1.159015	1159015	L (76)	15
			20.0	1.158987	1158987	- (10)	35
			21.0	1.158947	1158947	N (78)	25
			22.0	1.158932	1158932	E (69)	1
			23.0	1.158904	1158904	T (84)	26
1.7	2.7	3.7	24.0	1.235119	1235119	W (87)	16
			25.0	1.235099	1235099	O (79)	26
			26.0	1.235079	1235079	R (82)	9
			27.0	1.235061	1235061	K (75)	22
			28.0	1.235044	1235044	S (83)	13

Ciphertext: 931322328182097371835161213

**Step 9**: The CTEXT is then forwarded to the DSS to get the encrypted signal.

**Step 10**: We can retrieve the CTEXT from the RSS based on the encrypted signal.

**Step 11**: The plaintext is recovered via the SCA decryption scheme and the PKEY, and the result is shown in Table 7.

**Table 7:** Decryption of ciphertext to plaintext.

Time *τ*_1_	Time *τ*_2_	Time *τ*_3_	Time *t*	y_3_(t)	Keys $K_{i}$	Ciphertext ${C_{T}}_{i}$	Plaintext ${P_{T}}_{i}={C_{T}}_{i}-K_{i}$ (mod 38)
1.5	2.5	3.5	14.0	1.089718	1089718	32	78 (N)
			15.0	1.088824	1088824	3	69 (E)
			16.0	1.088195	1088195	36	85 (U)
			17.0	1.088692	1088692	36	82 (R)
			18.0	1.088484	1088484	1	65 (A)
1.6	2.6	3.6	19.0	1.159015	1159015	15	76 (L)
			20.0	1.158987	1158987	35	10 (-)
			21.0	1.158947	1158947	25	78 (N)
			22.0	1.158932	1158932	1	69 (E)
			23.0	1.158904	1158904	26	84 (T)
1.7	2.7	3.7	24.0	1.235119	1235119	16	87 (W)
			25.0	1.235099	1235099	26	79 (O)
			26.0	1.235079	1235079	9	82 (R)
			27.0	1.235061	1235061	22	75 (K)
			28.0	1.235044	1235044	13	83 (S)

Ciphertext: 931322328182097371835161213

Message: NEURAL NETWORKS

### 6.1 Discussion on the effects of parameter, fractional-order and time-delay on chaos synchronization

The impacts of varying parameter values, fractional-order, and other types of time-delay on chaotic synchronization are discussed in this section. Table 2 summarizes some observations about dynamic error synchronization based on examples 1–3 for various parameter values, fractional-order, and different types of time-delay at specified time values. Because neurons have varied communication delays, we study the non-identical multi-FONNSMD with other parameters and various time-delays. Based on the literature, fractional-order influences the system’s performance in chaotic dynamics. As formerly proven, parameters and time-delay affect the system’s effectiveness in chaotic dynamics, as seen in Figs 7–9. Time-delays are prevalent and inevitable in the real world, while the time-delay dynamic system has complex characteristics. The effect of varied parameters, fractional-order, and time-delay on dynamic systems is investigated using two multi-FONNSMD chaotic systems that operate as DRS. In addition, the competence of the SMC is explored in this study to demonstrate that it can control the multi-FONNSMD system.

We investigate a non-identical multi-FONNSMD system with multiple time delays, as shown in Fig 7, with fractional-order values of 0.99, 0.98, 0.97, 0.96, and 0.95. We can learn from Fig 7(a) and 7(b) that the system becomes chaotic and periodic when the parameter A = P = diag [1 1 1]. The system keeps on being chaotic as the different parameter values are employed. For example, parameters A and P where A = diag [1 2 2] and P = diag [2 2 2] in Fig 8(c) and 8(d). Then, A = diag [10.5 10.5 0.5] and P = diag [20.5 20.5 20.5] in Fig 9(e) and 9(f). Other values of parameters show that the multi-FONNSMD systems are chaotic but unstable since the dynamic synchronization error does not converge to zero. This paper demonstrates that parameters do have an impact on the system’s chaos synchronization. Table 2 presents some data on the dynamic synchronization error for non-identical multi-FONNSMD systems based on examples 1 and 2. Tests are carried out with a specified fractional-order and time-delay value. Example 3 is a non-identical multi-FONNSMD system run with a specified fractional-order value and no time delay.

We chose the values for fractional-order 0.99, 0.98, 0.97, 0.96, and 0.95 because they are the most stable based on the previous study compared to other fractional-order values. Furthermore, when the alpha value is 0.95 or above, a fractional chaotic system will exhibit chaotic behaviours. We chose it and verified it using a Matlab test, finding that the dynamic synchronization error approximates zero when the systems are synchronized. Furthermore, the main goal of our paper is to show that there is chaos synchronization with varied values of parameters, fractional-order, and time-delay. Furthermore, we show that chaos synchronization happens using the suggested controller presented in the paper. Also, we use a time-delay of τ = [1.5, 2.5, 3.5] for calculation purposes. As long as the chaos feature does not deteriorate, the value can be anything. Due to the obvious number of possible possibilities or the size of the data, some problems are difficult for computers to answer. As a result, they were picked to solve them effectively and avoid errors in the outcomes.

## 7. Conclusion

The combination of the synchronizing of a chaotic neural network with a secure communication system has been presented. Besides, this paper demonstrates that the multi-FONNSMD can be easily controlled using SMC techniques. So, we showed synchronization between the two multi-FONNSMD using the proposed controller. The synchronization is achieved as the convergence of synchronization errors of finite time has been ensured with the initial conditions for DSS and RSS given as $x_{1}\left(0\right)=0.22,$ $x_{2}\left(0\right)=0.22,$ $x_{3}\left(0\right)=-0.22$, y_1_(0) = 0.22, y_2_(0) = 2.22, y_3_(0) = 2.1. We also performed a different type of delay to compare the results of dynamic errors. We can conclude that chaos synchronization is achieved with a different type of delay and without delay. This is proof that we could not avoid delays occurring in the systems. After synchronization is archived, the secret keys are generated to be used in the NNSE algorithm. This new NNSE algorithm will give the proposed idea high security and efficiency. Finally, a numerical simulation is presented to support the efficiency of our proposed technique. Moreover, in the future, we anticipate exploring NNSE using asymmetric encryption.

## Supporting information

S1 Table Time taken of error dynamics of to converge to zero for the FONNSMD system with multiple time-varying delay. (PDF)

S2 Table Time taken of error dynamics of to converge to zero for the FONNSMD system without delay. (PDF)

S1 File Matlab code of FONNSMD system. (PDF)
