# Supplementary material for: Implementation of synchronization of multi-fractional-order of chaotic neural networks with a variety of multi-time-delays: Studying the effect of double encryption for text encryption
Source: PLoS One. 2022 Jul 1;17(7):e0270402. doi: 10.1371/journal.pone.0270402 (PMC9249245; doi:10.1371/journal.pone.0270402)
Supplement: S1 Table — (PDF) [file pone.0270402.s001.pdf]

S1 Table.

t	Multi time-varying delay		
	$S_E(t)_1$	$S_E(t)_2$	$S_E(t)_3$
0.5	0.634659	0.598428	25.035786
1.0	0.627415	-0.116200	12.685434
1.5	0.957937	0.073853	3.561962
2.0	0.415925	-0.278293	-0.657083
2.5	0.413797	-0.072367	-0.790369
3.0	0.317203	0.024769	-0.136943
3.5	0.144009	-0.018906	0.194559
4.0	0.072215	-0.026739	-0.012102
4.5	0.055296	-0.006980	-0.041575
5.0	0.036039	-0.002471	0.003638
5.5	0.020828	-0.003567	0.012974
6.0	0.013330	-0.003177	0.004287
6.5	0.009848	-0.001921	0.001811
7.0	0.007353	-0.001336	0.003530
7.5	0.005492	-0.001184	0.003885
8.0	0.004313	-0.001028	0.003211
8.5	0.003544	-0.000853	0.002775
9.0	0.002963	-0.000726	0.002589
9.5	0.002498	-0.000637	0.002383
10.0	0.002126	-0.000560	0.002141
10.5	0.001820	-0.000491	0.001923
11.0	0.001558	-0.000430	0.001734
11.5	0.001328	-0.000377	0.001561
12.0	0.001124	-0.000328	0.001398
12.5	0.000942	-0.000284	0.001249
13.0	0.000777	-0.000244	0.001111
13.5	0.000626	-0.000206	0.000983
14.0	0.000488	-0.000172	0.000864
14.5	0.000361	-0.000140	0.000754
15.0	0.000244	-0.000110	0.000650
15.5	0.000135	-0.000082	0.000554
16.0	0.000034	-0.000056	0.000463
16.5	-0.000060	-0.000031	0.000378
17.0	-0.000148	-0.000008	0.000297
17.5	-0.000231	0.000013	0.000222
18.0	-0.000309	0.000033	0.000150
18.5	-0.000382	0.000053	0.000083
19.0	-0.000451	0.000071	0.000019
19.5	-0.000516	0.000089	-0.000041
20.0	-0.000578	0.000105	-0.000099
