# Supplementary material for: Implementation of synchronization of multi-fractional-order of chaotic neural networks with a variety of multi-time-delays: Studying the effect of double encryption for text encryption
Source: PLoS One. 2022 Jul 1;17(7):e0270402. doi: 10.1371/journal.pone.0270402 (PMC9249245; doi:10.1371/journal.pone.0270402)
Supplement: S2 Table — (PDF) [file pone.0270402.s002.pdf]

S2 Table.

t	Without delay		
	$S_E(t)_1$	$S_E(t)_2$	$S_E(t)_3$
0.5	0.033672	0.013998	-0.739776
1.0	0.075061	0.061704	-0.281366
1.5	0.055032	0.047897	-0.079238
2.0	0.033062	0.029699	-0.001114
2.5	0.017541	0.016185	0.022607
3.0	0.008163	0.007758	0.024123
3.5	0.003127	0.003102	0.018433
4.0	0.000745	0.000826	0.011951
4.5	-0.000191	-0.000115	0.006877
5.0	-0.000433	-0.000394	0.003542
5.5	-0.000394	-0.000392	0.001606
6.0	-0.000277	-0.000301	0.000604
6.5	-0.000165	-0.000204	0.000153
7.0	-0.000084	-0.000130	-0.000010
7.5	-0.000033	-0.000081	-0.000043
8.0	-0.000005	-0.000053	-0.000026
8.5	0.000007	-0.000037	0.000000
9.0	0.000012	-0.000030	0.000023
9.5	0.000014	-0.000026	0.000037
10.0	0.000013	-0.000025	0.000045
10.5	0.000012	-0.000024	0.000049
11.0	0.000011	-0.000023	0.000049
11.5	0.000010	-0.000022	0.000048
12.0	0.000009	-0.000022	0.000046
12.5	0.000009	-0.000021	0.000044
13.0	0.000008	-0.000020	0.000043
13.5	0.000008	-0.000019	0.000041
14.0	0.000008	-0.000019	0.000039
14.5	0.000007	-0.000018	0.000038
15.0	0.000007	-0.000018	0.000037
15.5	0.000007	-0.000017	0.000035
16.0	0.000007	-0.000016	0.000034
16.5	0.000006	-0.000016	0.000033
17.0	0.000006	-0.000016	0.000032
17.5	0.000006	-0.000015	0.000031
18.0	0.000006	-0.000015	0.000031
18.5	0.000006	-0.000014	0.000030
19.0	0.000006	-0.000014	0.000029
19.5	0.000005	-0.000014	0.000028
20.0	0.000005	-0.000013	0.000028
