# Supplementary material for: Implementation of synchronization of multi-fractional-order of chaotic neural networks with a variety of multi-time-delays: Studying the effect of double encryption for text encryption
Source: PLoS One. 2022 Jul 1;17(7):e0270402. doi: 10.1371/journal.pone.0270402 (PMC9249245; doi:10.1371/journal.pone.0270402)
Supplement: S1 File — (PDF) [file pone.0270402.s003.pdf]

S1 File

Three programs, AfonnsMNOcontrol, FDE PI1 Ex, and fde12, must be written as M-files and then saved in the MATLAB software's work directory. The first program named AfonnsMNOcontrol is:

```
f_fun = @(t,y) [

 -  1*y(1)  +  0.2*tanh(y(1))   +  0.5*tanh(y(2))  +   5.5*tanh(y(3))   +
0.7*tanh(y(1)-1.5)  +      0.7*tanh(y(2)-1.5)   +   4.1*tanh(y(3)-1.5) ;
 -  1*y(2)  +  0.5*tanh(y(1))   +  0.5*tanh(y(2))  +   5.1*tanh(y(3))   +
0.1*tanh(y(1)-2.5)  +      0.1*tanh(y(2)-2.5)   +   2.5*tanh(y(3)-2.5) ;
 -  1*y(3)  -  0.5*tanh(y(1))   -  0.1*tanh(y(2))  -   0.5*tanh(y(3))   -
50.1*tanh(y(1)-3.5)  -    100.1*tanh(y(2)-3.5)   +   2.5*tanh(y(3)-3.5) ;

 -  1*y(4)  +  0.2*tanh(y(4))  +  0.3*tanh(y(5))  +    7.5*tanh(y(6))   +
0.3*tanh(y(4)-1.5) +      5.5*tanh(y(5)-1.5)   +   5.5*tanh(y(6)-1.5)     ;
 -  1*y(5)  +  0.5*tanh(y(4))  +  0.5*tanh(y(5))  +    7.5*tanh(y(6))   +
0.5*tanh(y(4)-2.5) +      2.5*tanh(y(5)-2.5)   +   5.5*tanh(y(6)-2.5)     ;
 -  1*y(6)  -  0.5*tanh(y(4))  -    3*tanh(y(5))  -    0.5*tanh(y(6))   -
50.1*tanh(y(4)-3.5) -     50.5*tanh(y(5)-3.5)   -   2.5*tanh(y(6)-3.5)

 ];


alpha = [1, 0.99, 0.98, 0.97,0.96, 0.95];
t0 = 0 ;
T =20 ;
y0 = [ -0.22 ; -0.22 ; -0.22 ; -0.22 ; -0.22 ; -0.22] ;
h = 0.005  ;
[t, y_f_fun]  = FDE_PI1_Ex(alpha,f_fun,t0,T,y0,h) ;

%% figure for synchronization of master system and slave system states
combne
subplot(2,2,1)
plot(t,y_f_fun(1,:)  ,'b')
hold on
plot(t, y_f_fun(4,:) ,'r')
xlabel ('t'), ylabel ('x_1 and y_1')
legend('x_1','y_1')

subplot(2,2,2)
plot(t,y_f_fun(2,:)  ,'b')
hold on
plot(t,y_f_fun(5,:)  ,'r')
xlabel ('t'), ylabel ('x_2 and y_2')
legend('x_2','y_2')

subplot(2,2,3)
plot(t,y_f_fun(3,:) ,'b')
hold on
plot(t,y_f_fun(6,:) ,'r')
xlabel ('t'), ylabel ('x_3 and y_3')
legend('x_3','y_3')


%% figure for synchronization errors(e1,e2,e3,e4) combine
```

```
subplot(2,2,4)
plot(t,y_f_fun(4,:)+ y_f_fun(1,:)  ,'b'  )
hold on
plot(t,y_f_fun(5,:)+ y_f_fun(2,:)  ,'r')
hold on
plot(t,y_f_fun(6,:)+ y_f_fun(3,:)  ,'g')
hold off
xlabel ('t'), ylabel ('e_1  e_2  e_3 ')
legend('e_1' , 'e_2' , 'e_3' )
```

For the second and third program, they are referring to [1]

[1] R. Garrappa, "Numerical solution of fractional differential equations: A survey and a software tutorial," Mathematics, vol. 6, no. 2, p. 16, Jan. 2018, doi: 10.3390/math6020016.
